# Muscle glycogen concentrations and response to diet and exercise regimes in Warmblood horses with type 2 Polysaccharide Storage Myopathy

**DOI:** 10.1371/journal.pone.0203467

**Published:** 2018-09-05

**Authors:** Zoë J. Williams, Megan Bertels, Stephanie J. Valberg

**Affiliations:** McPhail Equine Performance Center, Department of Large Animal Clinical Sciences, College of Veterinary Medicine, Michigan State University, East Lansing, Michigan, United States of America; University of Minnesota, UNITED STATES

## Abstract

Type 1 polysaccharide storage myopathy (PSSM1) is a glycogen storage disorder of known cause whereas the basis for type 2 PSSM (PSSM2) is unknown. The same diet and exercise regime prescribed for PSSM1 is recommended for PSSM2; however, the benefit of these recommendations for PSSM2 is undocumented. The objectives of this study were to determine traits of PSSM2 Warmblood horses (WB), determine the changes in exercise responses that occur with a recommended low-starch/fat-supplemented diet and exercise regime, and determine if glycogen concentrations correspond to the severity of signs. Owners of PSSM2 WB (2008–2016), completed a retrospective questionnaire regarding their horse. Glycogen concentrations were analyzed in skeletal muscle of PSSM2 WB (n = 36) obtained prior to recommendations and in control WB with no evident myopathy (n = 23). Chi-square, Fisher’s exact, McNemar’s tests with Bonferroni correction and Mann Whitney testing were utilized. Abnormal exercise responses reported by owners, began at approximately 6 years of age and included a decline in performance, a reluctance to collect and reluctance to go forward in over 50% of horses. With the recommended diet and exercise regime, 80% of PSSM2 WB owners reported an overall improvement with significant decreases in the proportion of horses showing a decline in performance and rhabdomyolysis. However, 53% of PSSM2 WB were still not advancing as expected with reluctance to go forward and collect persisting in approximately one third of horses. Median muscle glycogen concentrations did not differ between PSSM2 WB and WB with no evident myopathy. PSSM2 WB with the highest glycogen concentrations were significantly more likely to show a decline in performance than those with lower glycogen concentrations. In conclusion, diet and exercise recommendations ideal for PSSM1 improve but do not eliminate the decline in performance and reluctance to go forward under saddle characteristic of PSSM2.

## Introduction

In 1992, Polysaccharide Storage Myopathy (PSSM) associated with chronic ER was discovered in Quarter Horse-related breeds based on the hallmark feature of amylase-resistant polysaccharide in skeletal muscle biopsies [1]. Aggregates of amylase-sensitive glycogen and subsarcolemmal lakes of muscle glycogen were later added as potential diagnostic features which expanded the range of breeds affected by PSSM [2]. Sixteen years after the original discovery of PSSM, a dominant R309H gain of function mutation was identified in glycogen synthase 1 (*GYS1*) through a Quarter Horse mapping study that used phenotypic criteria of amylase-resistant polysaccharide and >1.5 fold elevations in muscle glycogen concentrations [3]. Analysis of over 830 horses of a variety of breeds in the US determined that 72% of Quarter Horses and 18% of WB diagnosed with PSSM by the presence of aggregates of amylase- sensitive or resistant glycogen in muscle samples possessed the *GYS1* mutation [4]. In the United Kingdom, 21% of horses diagnosed with PSSM by muscle biopsy were found to have the *GYS1* mutation [5]. Because some horses diagnosed with PSSM by muscle histopathology did not possess the *GYS1* mutation, two terms were applied; type 1 PSSM (PSSM1) was used to denote horses with the *GYS1* mutation and type 2 PSSM (PSSM2) to denote horses that had abnormal histologic muscle glycogen yet lacked the *GYS1* mutation. The specific cause or causes for PSSM2 remain unknown.

The management recommendations for PSSM provided to horse owners since 1997 have been similar for PSSM1 and PSSM2 and directed at reducing muscle glycogen synthesis, promoting oxidative metabolism and providing fat as an alternative fuel [6–8]. Recommendations include a diet that provides < 20% of digestible energy (DE) as nonstructural carbohydrate (NSC), 15–25% of DE as fat and regular daily exercise [9]. These recommendations were developed and found to be efficacious using a herd of Quarter Horses that had on average 1.8 times higher muscle glycogen concentrations than normal horses and, when tested in retrospect, had PSSM1 [9; 10]. The efficacy of the diet and exercise recommendations for PSSM has been evaluated in clinical cases through retrospective studies performed prior to identification of the *GYS1* mutation [11–13]. These studies, which likely included both PSSM1 and PSSM2 horses, found that in Quarter Horse-related breeds 100% of horses had a decrease in the frequency of ER and 71% had no further ER episodes [11]. In WB, clinical signs of PSSM also improved in 71% of PSSM horses [12]. Whether these improvements were specific to PSSM1 vs PSSM2 horses could not be ascertained from these early studies.

Recently, a retrospective study found that, unlike PSSM1, mean muscle glycogen concentrations in 13 PSSM2 WB horses were not above normal reference ranges [14]. If PSSM2 is not a glycogen storage disorder, it is possible that the current recommendations for low NSC, fat supplemented diets are not optimal for PSSM2 WB [9; 15]. To determine how well PSSM2 WB respond to current recommendations, we performed a retrospective study to determine the specific exercise responses and behaviors owners attributed to PSSM2 in WB and the perceived changes owners recognized following receipt of PSSM diet and exercise recommendations. We also determined muscle glycogen concentrations in the respondent’s horses prior to changing diet and exercise to determine whether horses had excessive glycogen storage and if there was a relationship between muscle glycogen and response to recommendations.

## Materials and methods

To determine the effect of diet and exercise regimes on the traits and exercise responses associated with PSSM2, a questionnaire was designed for horses diagnosed with PSSM2 by the Neuromuscular Diagnostic Laboratory (NMDL). Validation of the questionnaire was achieved using previously established principles [16, 17]. The questionnaire was designed with the purpose of describing the range of traits exhibited by PSSM2 horses and to determine if exercise responses improved or declined after institution of diet and management recommendations. The target group identified was owners of WB horses previously diagnosed with PSSM2 by muscle biopsies that had been evaluated by the Neuromuscular Diagnostic Laboratory. Questions used in previous epidemiological studies of equine myopathies were selected for the initial questionnaire and modified to elicit information on specific traits, exercise responses, diet and exercise regimens [11, 12]. The questionnaire was previewed and screened by two veterinarians, an equine nutritionist, and three horse owners to validate the content and clarity of the questionnaire and to allow for revisions before it was opened for responses. A pilot test was run on owners with PSSM2 affected horses of non-Warmblood breeds. The questionnaire was distributed to 109 owners of PSSM2 WB in June 2016 and responses were collected through October 2016. Signalment, traits, exercise responses, management and improvement or decline in responses was assessed by the owner comparing the horse’s condition before to after institution of PSSM management recommendations.

This retrospective study obtained informed consent of all participants prior to data collection and previously obtained muscle biopsies were analyzed. An Institutional Animal Care and Use Committee and Animal Use Form exemption was obtained for this study. Because the research goal of the study was to obtain information regarding the horse's response to exercise and diet management and not to perform research directed at investigating the owners of the horses, an Institutional Animal Care and Use Committee (IACUC) Administrator at Michigan State University did not deem it necessary to obtain Institutional Review Board approval.

### Case selection

Records of the NMDL database were searched to identify horses diagnosed with PSSM2 based on accumulation of aggregates of either amylase-sensitive or amylase-resistant polysaccharide. If PSSM2 horses did not have a negative R309H *GYS1* genotype on file, frozen muscle biopsies were retrospectively tested. Cases were selected from 2008 to 2016 to enhance accurate owner recall, to avoid overlap with a previous epidemiologic study of PSSM and to provide a minimum of 5 months for horses to have followed the diet and exercise recommendations [12]. Out of 452 PSSM2 cases, telephone, email or home addresses were on file for 260 horse owners and 155 additional cases had up to date contact information for veterinary clinics that was used to seek owner contact information.

To determine how common the behaviors and exercise responses were in the region where participants were located, participants were asked to identify another horse in their barn that had no previous history of a neuromuscular disorder or, if not in their barn, owned by a friend that had a horse in their geographic area. Horses in this baseline population were the same breed type, approximate age, gender and level of work as PSSM2 horses.

### Recommendations

Each PSSM2 horse owner had been provided with written recommendations included with the original muscle histopathology report which included minimizing stress, providing regular routines and maximizing daily turn out (S1 File). Dietary recommendations included a range of DE for maintenance to moderate exercise from 16.4–24.6 MCal/day and directions to provide less than 20% of DE as NSC, 15–20% of DE as fat and 697–836 g of protein per day. In 2014, additional recommendations for horses with PSSM2 included providing a prolonged warm up with long and low stretching, as well as rest periods during exercise to allow muscle relaxation. For those horses that exhibited muscle atrophy, the addition of an amino acid or protein supplement was recommended (S1 and S2 Files).

### Questionnaire

Telephone calls, emails, and postal delivery were used to encourage owners to answer a questionnaire with up to three solicitations per owner. A description of the study and an electronic link to a questionnaire were provided by email and, for non-respondents, in paper format. The questionnaire had closed ended questions with the option for additional written responses and included sections on signalment, results of PSSM1 genetic testing, general health history, performance history, lameness history, exercise responses, traits, diet before and after the diagnosis of PSSM2, exercise and turnout regimes before and after the diagnosis of PSSM2, and changes in traits and responses after diagnosis of PSSM2 (S3 File).

Owners of horses in the baseline group answered an abbreviated questionnaire that contained the same sections as the PSSM2 questionnaire, but omitted questions related to alterations made following diagnosis of PSSM2. Rather than provide information with regard to the time prior to the diagnosis of PSSM2, owners provided current information extending up to the year prior to answering the questionnaire (S4 File).

#### Signalment

Age, breed, gender, information on current ownership, relationship to the horse (owner/rider/trainer), length of relationship, and alive or deceased status of the horse was ascertained. If the horse was deceased or re-homed, participants were asked to answer questions with regard to the months preceding death or rehoming (S3 File).

#### History

The horse’s performance discipline, highest level of training or competition and any change in performance was ascertained. Additionally, appetite, body condition score (BCS) and breeding history were obtained. Appetite was scored on a Likert-scale from 1–5, with 1 defined as poor and 5 defined as excellent [18]. Pre-existing conditions of pituitary pars intermedia dysfunction, equine metabolic syndrome, shivers, stringhalt, equine protozoal myelitis (EPM), laminitis, colic, heaves, and gastrointestinal ulcers were reported along with any assessment of serum vitamin E status. The type and age of onset of abnormal exercise responses and behaviors relating to PSSM2, circumstances leading to these responses, frequency and severity were recorded (S3 File).

Participants were asked to indicate if a lameness was present within the 6 months prior to the muscle biopsy, if a lameness examination was performed by a veterinarian, which leg and structures were affected, the cause/diagnosis established for the lameness and whether a bone scan was performed. Participants described any treatments for lameness, if treatment resolved the lameness satisfactorily, and if the lameness still affects the horse’s performance or participant’s expectations of the horse (S3 File).

#### Diet

The PSSM2 WB horses’ diet prior to muscle biopsy and diet at the time of the questionnaire was ascertained from a list of commonly fed feeds such as fresh grass, dried forages, complete feeds, low starch ration balancers and supplements. Owners were asked to indicate changes made to the diet in terms of specific feeds, portion size, feeding frequency, feeding times, protein sources, supplements and pasture grazing. Additionally, participants were asked if they felt that the diet change helped improve abnormal exercise responses (S3 File).

#### Exercise

The number of days without work, the frequency and duration of work under saddle and turn out time prior to muscle biopsy (PSSM2) and upon receiving the questionnaire was documented. Extent and type of warm-up as well as number of breaks during a ride were recorded. Owners indicated if they changed the horse’s discipline or exercise regime after the PSSM2 diagnosis. Changes to the duration and intensity warm up and work under saddle, use of a long and low frame and amount of turn out were recorded. The participants’ impression of whether the horse improved was reported as well as the timeframe over which any improvements occurred (S3 File).

#### Overall improvement

Participants evaluated their horse’s overall change in performance as a percentage increase or decrease from the horse’s initial performance level prior to muscle biopsy. A list of abnormal exercise responses, traits and behaviors was provided and participants indicated if they saw improvement, no change or decline in those signs (S3 File). Open ended questions were also included to allow expansion on the severity and frequency of any current abnormal exercise or behavioral responses or further treatments.

### Muscle biopsy histopathology and glycogen concentration

#### Muscle histopathology

Staining of frozen muscle biopsy specimens at the time of submission included hematoxylin and eosin, modified Gomori trichrome, periodic acid Schiff (PAS), amylase-PAS and oil red O. Desmin immunohistochemistry was performed retrospectively. Sections from questionnaire respondent’s horses were re-evaluated and scored for the presence of internalized myonuclei, anguloid atrophy, inclusions in Gomori trichrome, subsarcolemmal glycogen, cytoplasmic PAS positive aggregates, amylase-resistant polysaccharide and desmin positive cytoplasmic aggregates. Findings were summarized using a previously described scoring system that used 20x magnification to grade the above features based on: 0 = not present, 1 = present in approximately 10% of fibers in the biopsy, 2 = present in approximately 11–25% of fibers in the sample, 3 = present in more than 25% of fibers in the sample [19]. A desmin score was assigned based on: 0 = not present, 1 = present in 1–2 fibers, 2 = present in 3–6 fibers, 3 = present in 7–10 fibers, 4 = present in >10 fibers [19]. The proportion of horses with a score > 1 was determined for each histopathologic characteristic except desmin where a score > 2 was used.

#### Muscle glycogen concentrations

Muscle glycogen concentrations were analyzed in those respondent’s PSSM2 WB horses that had enough frozen skeletal muscle tissue available for analysis (n = 32 shipped, 4 snap frozen out of 42 PSSM2 WB). Samples were either snap frozen in liquid nitrogen at the time of biopsy or shipped fresh on ice packs and received in the laboratory within 24–48 h. Fresh samples shipped in a similar fashion from 10 WB and 11 Quarter Horses with no histopathologic evidence of a myopathy and 14 WB and 7 Quarter Horses that possessed the R309H *GYS1* mutation were used as negative and positive controls, respectively for fresh shipped samples. Snap frozen samples from 9 healthy WB and 11 healthy Quarter Horses were used as controls for snap frozen samples. Glycogen concentrations were assayed fluorometrically as glucose residues using approximately 4 mg wet weight muscle as previously described [20].

### Statistical analysis

Responses were compiled in a spreadsheet [Microsoft Excel^®^], and the diagnosis of PSSM2 by the NMDL was verified by cross referencing NMDL records. Not all respondents answered each question so results were calculated relative to the total number of responses for each question. To determine whether exercise responses or behaviors responded to recommendations, responses for post-PSSM2 diagnosis were compared to answers for pre-PSSM2 diagnosis. Responses were analyzed with Chi-square and Fisher’s exact tests [GraphPad Prism 7^®^]. All paired continuous data were analyzed with a generalized McNemar’s test [21]. To reduce the risk of an alpha error, Bonferroni correction (p = 0.05/n) was performed for related responses. This included responses pertaining to exercise/performance (n = 4; p ≤ 0.013), traits associated with a neuromuscular disorder (n = 8; p ≤ 0.006) and behavior (n = 4; p ≤ 0.013). Skeletal muscle glycogen concentrations in shipped PSSM2 samples were not normally distributed (Shapiro-Wilk normality test) and median values were compared to PSSM1 and to NM using a Mann-Whitney U test [GraphPad Prism 7^®^]^2^. PSSM2 WB were divided into those with glycogen values above the median and those with glycogen values below the median. Above and below median glycogen groups were analyzed for differences in responses before and after the recommendations using Chi-square. Significance was set at p ≤ 0.05.

## Results

### Responses to questionnaire

In total, contact was made with 89 owners by email and for those that did not respond 20 surveys were mailed. A total of 42 completed questionnaires (38.5% response rate) were received from PSSM2 WB horse owners. WB was defined as horses identified as a type of Warmblood (n = 33), draft cross (n = 5) or WB cross (n = 4). Not all respondents answered each question and therefore the denominator varies in the results (S5 File).

### Baseline population

The baseline population consisted of 22 WB horses in the same region as PSSM2 WB. Horses were 13 ± 6 years of age with 6 mares, 15 geldings, and 1 stallion. Most were used for dressage (14/22, 63%) and hunter/jumper disciplines (11/22, 50%). The highest level of competition was Prix St. George for dressage and 1.1–1.5 meter for jumpers. Lameness was present in 27% (6/22) of WB in the baseline population within in the last year. All of those horses had a lameness exam by a veterinarian and 67% (4/6) had a forelimb and 33% (2/6) a hind limb lameness. Only 1 control horse had nuclear scintigraphy, for which the scan had positive uptake in a forelimb. The frequency of traits and exercise responses in the baseline WBs were; difficulty with canter transitions/leads (4/22; 36%), reluctance to go forward (3/22; 27%), generalized atrophy (2/22; 18%), mild lameness (2/22; 18%), poor topline (3/22; 27%), difficulty backing up (1/22; 9%), prolonged recumbency (1/22; 9%), sensitivity to grooming (1/22; 9%), bucking (1/22; 9%), and resentment to girthing (3/22; 27%) were reported (S6 File).

### PSSM2 WB

#### Signalment

At the time of the questionnaire, the 42 PSSM2 WB were 12 ± 6 y of age with 13 mares, 28 geldings, and 1 stallion.

#### Disciplines

Most PSSM2 WB were used for dressage (33/41, 80%) and hunter/jumper disciplines (6/41, 14.6%). The majority of horses competed at training (n = 5), first (n = 5) or second (n = 8) level dressage from United States Dressage Federation standards. The highest level of competition was Prix St. George for dressage and 1.1–1.5 meter for jumpers.

#### Time in respondent’s possession

Over 90% of the respondents were owners and ≤ 10% were either the trainer, primary rider, or primary caretaker. The majority of respondents had their horses for > 5yrs (64%). Most horses were alive at the time respondents answered the questionnaire (33/41, 80%); however, 20% (8/41) of PSSM2 WB were euthanized at some point after PSSM2 diagnosis.

#### History

Respondents reported a mean appetite score of 4.4 ± 0.9 and a mean BCS of 4.9 ± 1.3. A few horses had a history of colic (11/38, 29%), laminitis (2/39, 5%), pituitary pars intermedia dysfunction (1/41, 2%), heaves (0/39, 0%), equine metabolic syndrome (1/39, 2%), shivers (2/39, 5%), stringhalt (1/39, 2%), or equine protozoal myeloencephalitis (EPM) (2/39, 5%). Several PSSM2 WB have been diagnosed with gastric ulcers via endoscopy (13/36, 36%). Of the horses that pursued treatment(s), 88% (15/17) used omeprazole, 29% (5/17) used other treatments such as sucralfate or ranitidine, and 35% (6/17) used hindgut buffers. When asked if treatment of gastric ulcers improved any clinical signs that led to a muscle biopsy, 17% (4/23) said yes, 39% (9/23) said no, 35% (8/23) indicated that the GI ulcers did not occur at the same time as clinical signs of PSSM2 and 9% (2/23) were unsure.

Seventeen PSSM2 WB respondents indicated that serum vitamin E concentrations had been measured. Of those, 41% (7/17) had adequate levels (> 2ug/ml), 6% (1/17) had marginal levels (1.5-2ug/ml), 24% (4/17) were deficient (< 1.5ug/ml), and 29% (5/17) were unsure.

#### Lameness

Of horses affected by a lameness within the last 6 months, 47% (9/19) of PSSM2 WB still had lameness affecting the horse’s performance at the time of the questionnaire. Of those that had nuclear scintigraphy, 63% (5/8) had positive uptake and 37% (3/8) had a negative scan. Of the 5 PSSM2 WB with positive scans, 40% (2/5) had uptake in a forelimb, 60% (3/5) had uptake in a hind limb, 40% (2/5) had uptake in the neck, and 80% (4/5) had uptake in the hips or pelvis.

#### Onset of abnormal exercise responses

PSSM2 WB were first noted to have abnormal exercise responses related to PSSM2 at 6.4 ± 3.5 y of age with 48.8% (20/41) being ≤ 5y. The length of time over which owners perceived these abnormal responses developed was highly variable for PSSM2 WB, ranging from a few days or weeks (28%, 11/39), 1–2 months (31%, 12/39), 3 or more months (28%, 11/39) or unknown (13%, 5/39). Prior to the onset of abnormal exercise responses, 42% (16/38) of PSSM2 WB experienced a change in living situation, such as moving barns, and 37% (14/38) had a change in their diet. A consistent association with a weather pattern and these traits of PSSM2 was not reported.

#### Abnormal exercise responses

Before diagnosis of PSSM2 and recommendations, over half of the PSSM2 WB displayed abnormal performance related traits to include: decline in performance, reluctance to collect and a reluctance to go forward. General atrophy, difficulty with transitions, mild lameness, muscle fasciculations, poor topline, change in behavior, rhabdomyolysis, difficulty backing and resentment of saddling were only present in 30–48% of PSSM2 horses. The least common traits reported by PSSM2 respondents were bucking, sensitivity to grooming, prolonged recumbency and focal atrophy (Table 1).

**Table 1:** Traits, behaviors and exercise responses in a baseline WB population and PSSM2 WB alive at the time of questionnaire.

Clinical Signs	Baseline WB Population	PSSM2 WB		
	n = 22 (%)	Before Recommendations n = 33 (%)	After Recommendations n = 33 (%)	*P* value
**Performance**			**Significance adjusted *P* = 0.013**	
Reluctance to go forward	3 (27)	25 (76)	15 (45)	0.023
Decline in performance	0	22 (67)	7 (21)*	0.004
Reluctance to collect	0	19 (58)	10 (30)	0.046
Difficult canter transitions/ leads	4 (36)	15 (45)	11 (33)	0.450
**Neuromuscular**			**Significance adjusted *P* = 0.006**	
Generalized atrophy	2 (18)	16 (48)	5 (15)	0.007
Mild lameness	2 (18)	15 (45)	8 (24)	0.120
Muscle fasciculations	0	15 (45)	8 (24)	0.120
Poor topline muscle	3 (27)	15 (45)	10 (30)	0.310
Rhabdomyolysis	0	12 (36)	2 (6)*	0.005
Difficulty backing up	1 (9)	10 (30)	5 (15)	0.240
Prolonged recumbency	1 (9)	4 (12)	1 (3)	0.355
Focal area of atrophy	0	3 (9)	6 (18)	0.475
**Behavior**			**Significance adjusted *P* = 0.013**	
Overall change in behavior	0	12 (36)	3 (9)	0.017
Resentment of saddling/ girthing	3 (27)	10 (30)	6 (18)	0.389
Bucking	1 (9)	8 (24)	5 (15)	0.537
Sensitivity to grooming	1 (9)	7 (21)	4 (12)	0.511

*Significant differences comparing PSSM2 WB before and after recommendations.

#### Performance prior to PSSM2 diagnosis

A decline in performance was reported for 73% of PSSM2 WB, 27% of respondents noted that training had plateaued without advancing as expected and none felt performance was advancing as expected (Table 2).

**Table 2:** Respondent’s perception of the horse’s performance prior to the diagnosis of PSSM2 (before) and at least 5 months after receiving diet and exercise recommendations.

Performance	Before Recommendations	After Recommendations
	n (%)	n (%)
**Total number horses**	n = 41	n = 34
**Advancing**	0 (0)	16 (47)
**Not progressing**	11 (27)	12 (35)
**Declining**	30 (73)	6 (18)
**% decline**		
**1–25%**	7 (17)	1 (3)
**26–50%**	6 (15)	1 (3)
**51–75%**	6 (15)	0 (0)
**76–99%**	7 (17)	2 (6)
**100%**	4 (10)	2 (6)

### Muscle histopathology and glycogen concentrations prior to recommendations

#### Muscle histopathology

Histopathology scores ≥ 1 were found for: subsarcolemmal glycogen 50% (21/42); cytoplasmic glycogen 43% (18/42); anguloid atrophy 48%, (20/42); central nuclei 21% (9/42); subsarcolemmal vacuoles 12% (5/42); amylase resistant polysaccharide 5% (2/42). Desmin scores > 2, consistent with a potential myofibrillar myopathy, were found in 4/42 PSSM2 WB. Two of these horses were included in a previous study of myofibrillar myopathy [22].

#### Muscle glycogen concentrations

**Fig 1:**
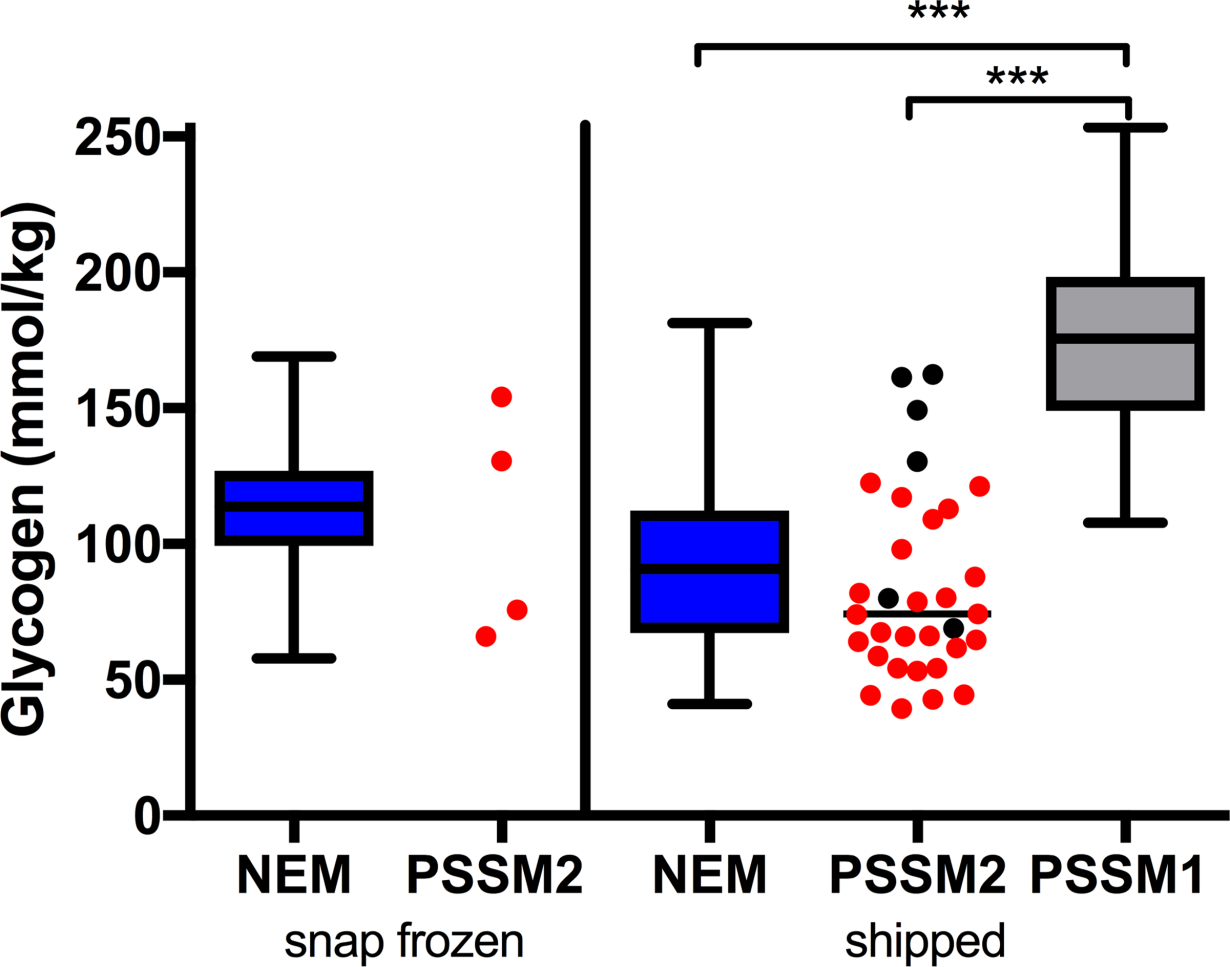
Skeletal muscle glycogen concentrations. Box and whisker plots indicate minimum to maximum values for snap frozen and shipped samples of control horses that had no evidence of a myopathy (NEM), type 1 PSSM (PSSM1), and individual values are shown for PSSM2. Black circles indicate euthanized WB horses. Glycogen concentrations were not different between PSSM2 WB and NM. PSSM1 glycogen concentrations were significantly higher than PSSM2 WB and NM horses (p < 0.0001).

A wide range in muscle glycogen concentrations was found for PSSM2 WB with some horses overlapping concentrations in PSSM1 horses and many falling below median values in NEM horses (Fig 1). Muscle glycogen concentrations in shipped PSSM2 WB samples were not significantly different from shipped NEM horses and were significantly lower (p < 0.0001) than PSSM1 horses (Fig 1 and S7 File). Four of the PSSM2 WB horses with the highest shipped glycogen concentrations were euthanized because of the severity of traits and one of the horses with the highest snap frozen glycogen was donated to a University due to the severity of abnormal exercise responses.

### Changes that occurred after PSSM2 diagnosis

#### Diet

After the PSSM2 diagnosis, 95% (39/41) of respondents felt they had changed their horse’s diets according to the recommendations provided. For those that changed their horse’s diet, 85% (33/39) of respondents reported that the diet had produced an overall improvement in exercise and behavioral traits. No improvement was reported for 13% (5/39) PSSM2 WB whereas 3% (1/39) were unsure if diet had made a difference.

In general, 87% (33/38) of PSSM2 WB respondents changed their horse’s sources of calories by increasing the amount of dietary fat fed, and/or increasing the amount of low starch/ fat supplemented concentrate (Tables 3 and 4). A change in protein intake was reported for 63% (24/38), dietary supplements were changed for 76% (29/38), and access to fresh grass was changed for 55% (21/38) (Tables 3 and 4). It was difficult to discern the precise diet changes that could have been associated with improved exercise and behavioral traits because some owners changed the diet prior to the diagnosis of PSSM2, most respondents did not have precise measures of the amount of various feeds being consumed and simultaneous changes in exercise regimes were adapted.

**Table 3:** Components of the diet of PSSM2 WB after recommendations were provided for a low starch, high fat diet.

Diet Components		After
		PSSM2 WB
**Hay**		**n = 41** **n (%)**
	Alfalfa	16 (39)
	Grass	39 (95)
**Fat Source**		**n = 36**
	Low starch + fat concentrate	15 (42)
	Solid	11 (31)
	Oil	16 (44)
	None	7 (19)
**Protein**		**n = 33**
	Alfalfa	17 (52)
	Amino acid supplement	7 (21)
	Ration balancer—20–30% protein	5 (15)
	No additional source	10 (30)
**Supplements**		**n = 27**
	Vitamin E (1000–6000 IU/day)	20 (74%)
	Magnesium	9 (33%)
	Vitamin & mineral supplement	9 (33)
	Acylcarnitine	7 (26)

**Table 4:** Changes owners made to their horse’s diet after receiving recommendations for PSSM2.

Diet Changes	PSSM2 WB (n = 37) n (%)
↓ grass hay fed	7 (19)
↑ grass hay fed	7 (19)
↓ alfalfa hay fed	8 (22)
↑ alfalfa hay fed	9 (24)
↓ fat supplement	2 (5)
↑ fat supplement	20 (54)
↓ complete feed (Equine Sr. Grain)	13 (35)
↑ complete feed (Equine Sr. Grain)	1 (3)
↓ low starch high fat concentrate	6 (16)
↑ low starch high fat concentrate	19 (51)
↓ low starch ration balancer	2 (5)
↑ low starch ration balancer	8 (22)
↓ time on fresh grass	12 (32)
↑ time on fresh grass	4 (11)

#### Exercise regime

After diagnosis, PSSM2 WB were ridden for 4 ± 2 days/week for a duration of 42 ± 11 min. Respondents reported that 53% (19/36) of WB were ridden for an average of 45 minutes, 25% (9/36) were ridden for and 22% (8/36) for 30 minutes or less. Half of the participants warmed their horse up for 11–15 minutes, 30% (12/40) warmed their horse up for 6–10 minutes, and the rest warm their horses up for 16–20 + minutes. The majority of respondents provide their horse with 3 or more breaks during a ride (33/38, 87%). Additionally, 70% of PSSM2 WB get some form of exercise every day and 61% (23/41) get actively worked under saddle 5 or more days a week.

#### Impact of changes to exercise regime

After diagnosis of PSSM2 and receipt of PSSM recommendations, 76% (31/41) of WB had their exercise regime changed and 24% (10/41) had no change. Of the changes made, 32% (10/31) of respondents increased the amount of time the horse was turned out, 42% (13/31) increased the frequency of exercise throughout the week and 13% (4/31) decreased both riding time and the level of intensity. In addition, 71% (22/31) of respondents increased the duration of their warm-up time, 65% (20/31) started to allow breaks throughout the ride and 67% (20/30) implemented long and low stretching techniques.

Of the PSSM2 WB respondents, 70% (21/30) felt that the change in exercise helped improve exercise responses, while 17% (5/30) saw no improvement, and 13% (4/30) were unsure if an improvement was present. Fifty-five % (11/20) of respondents that used long and low stretching felt it improve exercise responses while 45% (9/20) saw no difference. The time frame for general improvement was less than a month in 45% (10/22) of horses, 1–2 months in 18% (4/22), 3–4 months in 23% (5/22) and greater than 4 months for 14% (3/22).

#### Change in exercise responses and behavior

An overall improvement in responses and behavior was reported for 80% (32/40) of PSSM2 WB after receiving recommendations with the PSSM2 diagnosis. When comparing the responses after diagnosis and recommendations to before diagnosis for horses alive at the time of the questionnaire, PSSM2 WB were less likely to experience a decline in performance (p = 0.004) and rhabdomyolysis (p = 0.005) (Table 1). Although the traits of reluctance to collect, reluctance to go forward, overall change in behavior, and generalized muscle atrophy were also reported to improve they did not reach significance after Bonferroni correction (Table 1).

#### Change in performance

With the changes instituted after PSSM2 diagnosis, 47% of PSSM2 WB respondents reported that training was advancing as expected, 35% reported that performance had plateaued and not advance as expected and 18% reported that performance had declined (Table 2). Because additional recommendations of adding an amino acid supplement and using long and low warm up techniques were added in 2014, we analyzed the overall response of those with the additional recommendations compared to those without. For those without the additional recommendations, 18% (3/17) of respondents reported a decline in performance, 29% (5/17) indicated no advancement, and 53% (9/17) were reported to be advancing as expected. For those receiving the additional recommendations, 18% (3/17) reported a decline, 41% (7/17) indicated no advancement, and 41% (7/17) were reported to be advancing as expected. There was no significant difference in the respondent’s perception of the horse’s overall improvement with respect to the additional recommendations made in 2014.

Five of 31 PSSM2 WB respondents (16%) changed the horse’s discipline(s) after diagnosis and recommendations: 2 horses changed from dressage to hunter/jumper, 2 horses from dressage to trail and pleasure riding, and 1 dressage horse was retired. At some time point after PSSM2 diagnosis, 22% (9/41) of PSSM2 WB were euthanized (n = 8) or donated to a University (n = 1). Respondents indicated euthanasia was due to severe traits of PSSM2 (n = 5; 1 with concurrent gastric ulcers and laminitis), epilepsy (n = 1), neurologic disease (n = 1) or prolonged recumbency (n = 1).

#### Behavior and exercise responses relative to muscle glycogen concentrations

A decline in performance was reported for PSSM2 WB in both above and below median glycogen groups, however, a significantly larger proportion of horses above the median glycogen concentration had a decline in performance before diagnosis compared to those below the median glycogen concentrations (p = 0.005) (Table 5). All participants that had horses that were deceased at the time of the questionnaire answered questions describing exercise responses and traits before diagnosis (n = 5 above; n = 1 below median glycogen concentration). Only one participant with a deceased horse (above median glycogen) answered questions regarding responses and traits after recommendations. After the recommendations, the proportion of horses with a reported decline in performance significantly decreased in the above and below median glycogen groups (p = 0.0002, p = 0.006, respectively) (Table 5). Sixty-nine versus 30% of owners reported an overall change in behavior before diagnosis in the above vs. below median glycogen concentration groups, respectively. After recommendations, there was a significant decrease in the number of owners reporting a change in behavior in the above median glycogen group and no horse owners reported a change in behavior in the below median group after the recommendations (p = 0.009) (Table 5).

**Table 5:** Exercise responses and traits noted in at least 30% of horses before the diagnosis of PSSM2 and after diet and exercise recommendations subdivided into those horses with glycogen concentrations above the median glycogen concentration and those horses below the median glycogen concentration.

Clinical Signs	Glycogen above median		Glycogen below median	
	Before Diagnosis	After Recommendations	Before Diagnosis	After Recommendations
	n = 16 (%)	n = 12 (%)	n = 20 (%)	n = 16 (%)
**Performance**	**Significance adjusted p = 0.013**			
Decline in performance	16 (100%)^†^	4 (33%)*	12 (60%)	2 (13%)*
Reluctance to collect	11 (69%)	7 (58%)	11 (55%)	3 (19%)
Reluctance to go forward	15 (94%)	6 (50%)	13 (65%)	7 (44%)
**Neuromuscular**	**Significance adjusted p = 0.006**			
Generalized atrophy	9 (56%)	3 (25%)	7 (35%)	1 (6%)
Rhabdomyolysis	7 (44%)	1 (8%)	9 (45%)	1 (6%)
Muscle fasciculations	6 (38%)	4 (33%)	9 (45%)	3 (19%)
**Behavior**	**Significance adjusted p = 0.013**			
Overall change in behavior	11 (69%)	2 (17%)*	6 (30%)	0 (0%)

* indicates significant difference within the above or below median glycogen groups between before and after.

^†^ indicates a significant difference between the above and below median glycogen groups for the corresponding traits in the same time frame.

## Conclusions

The results of the present study provide a comprehensive owner reported description of the characteristics of PSSM2 in WB and for the first time provide valuable prognostic information with regard to response of PSSM2 WB to current diet and exercise management as perceived by owners. The onset of abnormal exercise responses and behaviors in PSSM2 WB was insidious with an average age of onset of 6 yrs, an age when WB horses are expected to be advancing in their training [23]. The three most common complaints of respondents owning PSSM2 WB were reluctance to collect under saddle, decline in performance, and reluctance to go forward reported by 58%, 67%, 76% of respondents, respectively. These exercise responses were uncommon in the baseline WB population at 0–27%. Respondents also reported that 6–24% of PSSM2 WB had mild lameness, muscle atrophy, muscle fasciculations and intermittent rhabdomyolysis. No horses in the baseline population had fasciculations or rhabdomyolysis whereas 18% had lameness (largely forelimb) or atrophy. Other traits and responses common to both PSSM2 WB and the baseline group included difficulty with canter transitions/leads, poor topline, sensitivity to grooming, resentment to girthing and bucking with frequencies ranging from 9–36% in baseline and 12 to 33% in PSSM2 WB. In a previous study of PSSM in WB and non-WB breeds, which included 25 WB in the present study, veterinarians reported that 66% of 188 PSSM2 WB horses had a gait abnormality and 26% had rhabdomyolysis [14]. To provide a more thorough description of the gait abnormality, participants in the present study were given detailed questions about their horse’s gait and lameness. The results revealed that the gait abnormality in PSSM2 WB consisted largely of an undiagnosed lameness that did not resolve with veterinary treatment in 60% of PSSM2 WB. Supplementing the finding of a gait abnormality by Lewis *et*. *al*. 2017, our results indicate that reluctance to go forward and to collect are prominent features of PSSM2 in WB which significantly impact the horse’s performance.

The lameness and decline in performance in PSSM2 WB described in the present study may overlap with signs associated with orthopedic disorders. In particular, sacroiliac disease is characterized by poor development of epaxial muscles, asymmetric hind limb muscles, stiffness, unwillingness to work on the bit and poor quality canter [24; 25]. Scintigraphic evaluation is commonly used to diagnose back and sacroiliac disease and was performed in 8 horses in the present study [26]. Of those 8 PSSM2 WB, 4 scans had positive results, but only 1 respondent indicated that their horse had been diagnosed with sacroiliac disease; however, the 3 remaining horses had reported positive uptake in the pelvic area. The overlap in clinical signs and common occurrence of sacroiliac disease in WB certainly suggests that diseases of the back and sacroiliac joint should be ruled out prior to performing a muscle biopsy. Additionally, a wide range of training and saddling issues could also produce many of the performance-related signs seen in PSSM2 horses and it seems prudent to fully explore these issues prior to a muscle biopsy [27].

Fortunately, by following the recommended diet and exercise regimes, 80% of PSSM2 WB were reported to show overall improvement as well as a significant decrease in signs of rhabdomyolysis, muscle atrophy, change in behavior and decline in performance. This is in agreement with a 2007 epidemiologic study of WB diagnosed with PSSM that reported 71% of WB with PSSM (type 1 or type 2 not specified) improved with recommendations for a low starch, fat supplemented diet and regular exercise [12]. The reported improvement is also higher than the placebo effect found in a caregiver study in which 40% of owners of dogs with osteoarthritis reported a beneficial effect when they unknowingly administered a placebo [28]. It is important to recognize, however, that although abnormal exercise responses and behaviors improved, one third of respondents felt that their horse’s training had plateaued and 18% felt their horse’s performance had declined after implementing recommendations. The most persistent traits were reluctance to go forward (45% still present), difficulty with transitions (33% still present), reluctance to collect (30% still present), poor topline muscle (30% still present) and muscle fasciculations (24% still present). Thus, owners of PSSM2 WB should be aware that although the majority of horses improve with the current diet and exercise recommendations, half of the horses are unable to achieve the owner’s performance expectations. Sixteen percent of respondents in the survey changed the horse’s discipline to one requiring less collection and 22% of PSSM2 WB were eventually euthanized or donated to a University. Our results suggest that even when following the current diet and exercise recommendations, there are residual effects of the muscle disorder underlying PSSM2. Potentially, this study’s results may have been biased since owners of more severely affected horses could have been more motivated to participate in the study.

With the present study design, there was no means to determine how stringently recommendations were followed by horse owners or what specific diet changes were responsible for the perceived improvement amongst horse owners. The most consistent changes in diet made by owners of PSSM2 WB were the addition of a fat supplement in 54% of horses, use of a low starch high fat feed in 51% and supplementation with vitamin E in 71% of PSSM2 WB. Generally, the dietary recommendations may have benefited horses because owners sought nutritional advice ensuring that horses received a balanced diet that was relatively low in starch and sugar, supplemented with fat and contained a quality protein source and vitamin E. Beginning in 2014, recommendations for PSSM2 horses included adding a protein source or amino acid supplement to build topline muscles and providing long and low warm up exercise to relax the back and strengthen topline. Provision of amino acid supplements has been shown to increase muscle mass in exercising horses as assessed by subjective muscle scoring [29]. However, there was no significant difference in performance when comparing horses that received recommendations before 2014 to those after–as shown by 50 vs 44% of PSSM2 WB horses advanced in their training, respectively. A clearer understanding of the basis for PSSM2 in WB and design of controlled diet trials and are needed to optimize diet and exercise regimes for these horses.

The diet and exercise regime recommended for PSSM2 horses was originally designed to decrease glycogen synthesis in skeletal muscle and improve oxidative metabolism of glycogen and fat during exercise. In PSSM1 horses, the diet effectively lowers serum insulin concentrations which could thereby decrease glucose uptake and decrease activation of glycogen synthase in skeletal muscle [9; 30]. Whereas glycogen concentrations are >1.5 fold normal in PSSM1 WB, a recent study did not find significantly elevated mean glycogen concentrations in the 13 PSSM2 WB studied [14]. Similarly, glycogen concentrations in the present study were not significantly different from horses with no evidence of a myopathy in muscle samples and were lower than PSSM1 horses. However, there was a large variation in glycogen content in PSSM2 WB both in snap frozen and shipped samples. Glycogen continues to be metabolized during shipping, and low levels could be a reflection of continued metabolism; however, control samples were also shipped in a similar fashion. It is possible that the horses with elevated muscle glycogen concentrations represent the extremes within one phenotype embodied by a disorder of glycogen metabolism. Most WB horses diagnosed with PSSM2 had very similar clinical signs and glycogen storage disorders in humans can have variable muscle glycogen concentrations [31]. Alternatively, those PSSM2 WB with glycogen concentrations as high as PSSM1 could represent a group of horses with a separate disease from horses with lower glycogen concentrations. The histopathologic criteria for PSSM2 are nonspecific which increases the possibility that more than one etiology for muscle disease falls under this histopathologic diagnosis. Four horses were later found to have histochemical characteristic of abnormal aggregates of desmin within muscle biopsies suggestive of myofibrillar myopathy (MFM) [22]. This finding again could represent progression of the PSSM2 phenotype or could represent a separate disease entity. Determining whether horses within the PSSM2 diagnosis have one etiopathology, or separate etiologies, will require further biochemical and genetic analyses.

An unexpected finding in the present study was the high prevalence of gastric ulcers (32%) confirmed by endoscopy in the PSSM2 WB horses. This is slightly lower than the prevalence of squamous ulceration (40%) reported in Warmblood show jumping horses [32]. It is unlikely that gastric ulcers themselves accounted for clinical signs of PSSM2 in WB horses because many of the horses had been effectively treated for ulcers yet performance problems persisted. The higher prevalence of ulcers in the PSSM2 WB could be related to high exercise frequency and performing at competitions; information that was unfortunately not obtained in our study [33].

The authors acknowledge the limitations of the present study, particularly that the results of the questionnaires are based on the perceptions of owners with varying levels of experience, and not those of a veterinarian.

In summary, the results of the present study indicate that PSSM2 in WB has a strong impact on performance affecting a horse’s willingness to collect and go forward. By following diet and exercise recommendations, exercise responses appear to improve in 80% of horses; however, 30–45% of horses have residual signs that impacted the horse’s ability to advance in training. While a small proportion of PSSM2 WB had elevated muscle glycogen concentrations, the majority of PSSM2 WB had concentrations at or below concentrations in horses without histopathologic evidence of a myopathy.

## Supporting information

S1 File Neuromuscular Diagnostic Laboratory Polysaccharide storage myopathy. Recommendations.
(PDF)

S2 File Type 2 polysaccharide sorage myopathy. Recommendations.
(PDF)

S3 File Neuromuscular Diagnostic Lab: Muscle biopsy follow-up survey. Questionnaire distributed to participants of PSSM2 WB.
(PDF)

S4 File Equine Neuromuscular Diagnostic Lab: Healthy performance horse questionnaire. Questionnaire distributed to baseline WB participants.
(PDF)

S5 File PSSM2 questionnaire data. All answers from PSSM2 questionnaire with anonymity.
(XLSX)

S6 File Baseline questionnaire data. All answers from baseline questionnaire with anonymity.
(XLSX)

S7 File Skeletal muscle glycogen concentrations. (XLSX)
