# Supplementary material for: Muscle glycogen concentrations and response to diet and exercise regimes in Warmblood horses with type 2 Polysaccharide Storage Myopathy
Source: PLoS One. 2018 Sep 5;13(9):e0203467. doi: 10.1371/journal.pone.0203467 (PMC6124783; doi:10.1371/journal.pone.0203467)
Supplement: S1 File — Recommendations. (PDF) [file pone.0203467.s001.pdf]

Neuromuscular Diagnostic Laboratory

Polysaccharide Storage Myopathy

What Causes PSSM?
Horses with polysaccharide storage myopathy (PSSM) accumulate excessive amounts of the normal form of sugar in their muscles (glycogen) as well as an abnormal form of sugar (polysaccharide) in muscle tissue. Clinical signs of are usually those of tying-up where horses develop muscle stiffness, soreness and reluctance to work with exercise. However, signs found in Draft, Draft crossbreeds, include muscle atrophy, weakness and gait abnormalities. Some horses with PSSM that are managed well show no clinical signs. Type 1 PSSM is caused by an inherited defect in a gene called glycogen synthase that results in abnormal regulation of glycogen in skeletal muscle. This appears to disrupt energy metabolism. We can now perform a genetic test for PSSM if you did not do so when the biopsy was submitted. You can request this test from hair or blood samples. Forms are on our website http://www.vdl.umn.edu/vdl/ourservices/neuromuscular.html We do not know yet what the defect is that causes type 2 PSSM.

More information regarding PSSM can be found at the Neuromuscular Disease Laboratory website http://www.cvm.umn.edu/umec/lab/home.html. We are unable to discuss individual cases with horse owners by telephone. We are pleased to discuss cases with your veterinarian. If owners of horses we have evaluated have questions please email or mail them to us. Email address is NMDL@umn.edu

Management of PSSM
Signs of muscle pain, atrophy and stiffness in horses with both types of PSSM can be managed through specific diet and training regimes. Both diet and training must be changed to see a beneficial effect. The diet is altered to minimize starch and sugar content. This means eliminating sweet feed, corn, wheat, oats, barley, and molasses. Alternative calories are supplied in the form of fat. An equally important part of PSSM horse management is daily exercise. Consistent exercise enhances glycogen utilization, increases enzymes needed to burn fat and improves energy metabolism in skeletal muscle. To burn fat efficiently, horses need a gradual training program in addition to more fat in their diet. Approximately 90% of horses experienced fewer or no episodes of tying-up if the recommendations provided below are followed. At present, the best we can do is to use management techniques in order to reduce recurrence. Many horses with this muscle disorder have recurrent episodes of tying-up.

1) Exercise recommendations
Provide daily turnout: Provide daily turn out for as long as possible with other horses to keep the horse active. Decrease the amount of time the horse is left in the stall as much as possible.

Starting exercise: If the horse has suffered from stiffness within the last two weeks, or has just been diagnosed as having PSSM, start by turning the horse out for two weeks while implementing the recommended dietary changes. Once the horse has been on the diet for two to four weeks, start round pen or light lunge line work once a day for three minutes at a walk and trot. This initial work should be very mild and very short in duration. Gradually increase the time in work by two minutes each day, with a two minute walk break before the increasing the exercise duration. If the horse seems stiff at any time, slow to a halt and rest for a minute if the stiffness persists, stop there; if not, continue after a two-minute walk. If the horse seems tired, do not continue to increase the work load, but let the horse become acclimated to the workload in an even more gradual manner.

When the horse can exercise for 15 minutes, provide a five-minute break at a walk, and then gradually increase walking and trotting for longer periods. At least three weeks of groundwork will precede any mounted exercise. When the horse can comfortably walk and trot for 30 minutes on a lunge line, you may start riding the horse at the same exercise intensity, starting with 20 minutes of combined walk/trot. Gradually increase the time the horse is ridden using intervals of walk and trot and then work into walk, trot, and canter. Be sure to monitor the horse's level of exertion. Your patience will be rewarded.

Maintaining exercise: Try to exercise the horse on a daily basis even if only for 15 minutes on a lunge-line. Research has consistently shown that even 10-15 min a day will improve the function of muscle in PSSM horses. Once your horse is fit it may well manage with turn out rather than daily exercise from time to time. The number of days off a horse can manage is highly individual. If more than 3 or 4 days have gone buy begin with a small amount of exercise.

2) Dietary Changes for PSSM Horses
Scientific evidence proves that it is **not necessary to feed 1 lb of fat a day** as you may see recommended on the internet. Excessive dietary fat will lead to obesity and metabolic syndrome. Judge the number of calories your horse needs first and adjust the diet accordingly.

Forage
A high-quality grass or oat hay should form the basis of the diet. If alfalfa hay must be fed, combine it with another low sugar hay like Bermuda grass hay or timothy. Vitamin and mineral supplements containing Vitamin E and selenium are beneficial; however, some feeds (including some recommended below) contain enough selenium and other vitamins and minerals and do not require additional supplements. Check with the feed company if there are any questions.

Electrolytes
Ensure that salt is always available. If horses will not use a salt block, add 1-3 tablespoon of loose iodized table salt in the feed, particularly in hot weather. If the horse is sweating a great deal, an additional tablespoon of lite salt (containing potassium chloride) can be added.

Complete Feeds
In my experience it is easiest to provide a balanced ration if a complete feed is fed. These feeds do not require additional protein/vitamin/mineral supplements and are to be fed along with hay. No additional grain or mineral/vitamin mix should be added.

Re-Leve®** by Hallway Feeds (www.Re-leve.com) Phone 1-800 753-4255
In Minnesota, **Re-Leve** is carried by Assurance Feeds Phone (651) 463-8041.
Developed with University of Minnesota researchers, **Re-Leve** is the only feed proven to be effective for PSSM and is good for finicky eaters. Starch content is low (9.0% by weight) and fat content high (12.5% by weight). Additional selenium should not be fed.

- **Two forms of Re-Leve exist.**
  - **Re-Leve original is for hard keepers needing more concentrate to maintain body weight.**
    - Feed 8-10 lbs for thin horses or horses in heavy work
  - **Re-Leve Concentrate is best for many PSSM horses that are easy keepers**
    - 3-5 lbs fed for light to moderate work

  For overweight horses, work with your veterinarian and avoid excessive fat supplementation. One approach would be to reduce hay to 1% of body weight and feed 1-3 lbs of **Re-Leve Concentrate**.

  For growing horses:
  - Weanlings: 6.5 lbs Re-Leve® and a mixed grass/alfalfa hay (8lbs per day)
  - Yearlings: 8 lbs of Re-Leve® and a 50-50 alfalfa/grass hay (9 lbs per day).

(**a portion of the proceeds from the sale of Re-Leve® are directed to Dr. Valberg)

Ultium® by Purina in the USA: www.purinamills.com. Or Phone 800-227-8941
- 6-8lbs per day

Safe Choice® by Nuterna. www.nutrenaworld.com.
- 6 lbs per day combined with **Empower®** at 2 lbs/day

UnTi available in Canada is a low starch and high fat feed

There are now several other low starch and high fat feeds available. Please speak to a nutritionist regarding these feeds. The quality of ingredients may vary so make sure you are working with a reputable company. At a minimum they should meet the nutritional requirements provided in Table 1. In general, the starch content of the feed should not be greater than 15-20% by weight and the fat should be greater than 10% by weight.

Blending of individual feeds
Fat supplements combined with additional protein/ vitamin /mineral mixes and a fiber base can be custom blended. You may find in the end that this is not a cost saver. Consult with the manufacturer's nutritionists to formulate the correct blend for a PSSM horse which is specific to breed and level of use using the values in Table 1.
Stabilized rice bran fat supplements:
EquiJewel® Kentucky Equine Research, Phone 859 873 1988, Fax 859 873 3781, Email info@ker.com. ker.com/supplements/Equijewel.html

Natural Glo® (rice bran) Alliance Nutrition. Phone 1-800 680-8254 email AM_EquineHelp@admworld.com, website www.admani.com/Alliance Equine/

Ultimate Finish® by Buckeye Nutrition, PO Box 505, 330 E. Schultz Ave. Dalton, OH 44618. Phone: 1-800-898-9467 Fax: (330) 828-2309, www.buckeyenutrition.com/equine

Vegetable Oils: Soy oil or Corn oil gradually can be added at 1-2 cups per day to a fiber base such as hay cubes or alfalfa pellets. Add 600 U of vitamin E/cup of oil per day.

Remember to weigh the feeds using a scale and not use volume to determine actual weight.

Table 1. Nutritional requirements for an average sized horse (500 kg /1100 lbs) with PSSM at varying levels of exertion*. Note NSC refers to the soluble sugar + starch. Fructans in forage are not considered in this calculation as they are not considered likely to impact the glycemic index.

	Maintenance	Light Exercise	Moderate Exercise	Intense Exercise
**Digestible Energy (DE) (Mcal/day)**	16.4	20.5	24.6	32.8
**% DE as NSC**	<20%	<20%	<20%	<20%
**% DE as fat**	15%	15%	15%-20%	20-25%
**Forage % bodyweight**	1.5- 2 %	1.5- 2 %	1.5- 2 %	1.5- 2 %
**Protein (g/day)**	697	767	836	906
**Calcium (g/day)**	30	33	36	39
**Phosphorus (g/day)**	20	22	24	26
**Sodium (g/day)**	22.5	33.5	33.8	41.3
**Chloride (g/day)**	33.8	50.3	50.6	62
**Potassium (g/day)**	52.5	78.3	78.8	96.4
**Selenium (mg/day)**	1.88	2.2	2.81	3.13
**Vitamin E (IU/day)**	375	700	900	1000

***** Disclosure of financial interest: Drs. McCue, Mickelson and Valberg are the patent owners for the genetic testing for *GYS1*. A portion of the proceeds from this test will go towards their continued research as well as patent royalties.

Although the type 1 PSSM (*GYS1* mutation) is the most common genetic cause of PSSM and tying up in Draft and Quarter Horse related breeds there are other causes of tying-up. If horses affected with chronic muscle diseases are negative for type 1 PSSM we recommend follow up with a muscle biopsy to investigate other possible causes, see http://www.cvm.umn.edu/umec/lab/home.html, for information on obtaining and submitting a muscle biopsy to the Neuromuscular Diagnostic Laboratory.
