# Supplementary material for: Muscle glycogen concentrations and response to diet and exercise regimes in Warmblood horses with type 2 Polysaccharide Storage Myopathy
Source: PLoS One. 2018 Sep 5;13(9):e0203467. doi: 10.1371/journal.pone.0203467 (PMC6124783; doi:10.1371/journal.pone.0203467)
Supplement: S2 File — Recommendations. (PDF) [file pone.0203467.s002.pdf]

Neuromuscular Diagnostic Laboratory
Michigan State University, College of Veterinary Medicine
Dr. Stephanie J. Valberg

Type 2 Polysaccharide Storage Myopathy

Terminology: PSSM, EPSM and EPSSM are terms first used to describe horses that have abnormal appearing glycogen in muscle biopsies. Considerable controversy existed as to whether these acronyms encompassed one muscle condition or several muscle conditions. In 2008, a mutation in the glycogen synthase 1 gene was found to be highly associated with one form of excessive storage of polysaccharide in muscle. Genetic testing of hundreds of horses previously diagnosed with PSSM showed that not all horses diagnosed with PSSM have this genetic mutation. This suggested that there are at least two forms of PSSM. For clarity, the form of PSSM caused by a glycogen synthase 1 (*GYS1)* gene mutation is now termed **type 1(PSSM1)** whereas the form of PSSM that is not caused by the *GYS1* mutation and whose origin is yet unknown is now termed **type 2 (PSSM2)**. PSSM1 is likely the same disorder described as "Azoturia " or "Monday Morning Disease" in work horses in the 19th and 20th centuries. We believe that there are probably several causes of type 2 PSSM.

Type 2 PSSM

There is much less known about type 2 PSSM, because as it turns out, previous research on PSSM has largely involved horses with type 1 PSSM. Current knowledge of type 2 PSSM is based on retrospective evaluation of cases diagnosed with PSSM by muscle biopsy that are now known be free of the *GYS1* mutation and a few years of prospective clinical cases.

Prevalence: Approximately 28% of cases of PSSM diagnosed by muscle biopsy in Quarter Horses do not have the *GYS1* mutation. Type 2 PSSM seems to be more common in higher performance horses such as barrel racing, reining and cutting horses compared to the high prevalence of type 1 PSSM in halter horses. About 80% of cases of PSSM diagnosed by biopsy in Warmbloods have type 2 PSSM. Breeds affected include Dutch Warmbloods, Swedish Warmbloods, Hanoverians, Friesians, Selle Francais, Westfalian, Canadian Warmblood, Irish Sport Horse, Gerdlander, Husien, and Icelandic horses. Many other light breeds have also been diagnosed with type 2 PSSM including Morgans, Arabians, Standardbreds and Thoroughbreds.

Pathophysiology: The cause of type 2 PSSM is currently unknown. It may well be that there are a group of conditions that have separate causes but share common findings of glycogen accumulation and poor performance. A heritable predisposition is suspected in Quarter Horses but yet to be proven. Recent research does not support higher than normal muscle glycogen concentrations in muscle from horses with type 2 PSSM. The glycogen aggregates within the cells but the total amount is not increased as in type 1 PSSM.

Acute Clinical signs: Horses with type 2 PSSM do not necessarily have the same calm temperament as horses with type 1 PSSM. In adults, acute clinical signs of rhabdomyolysis are similar between type 1 and type 2 PSSM. Muscle atrophy after rhabdomyolysis is a common complaint in Quarter Horses with type 2 PSSM and this may not be preceded by exercise. There are more Quarter Horses less than one year of age reported with type 2 PSSM than type 1 PSSM and these foals may present with an inability to rise or a stiff hind limb gait.

Chronic clinical signs: Chronic signs of type 2 PSSM are often most closely related to poor performance rather than recurrent ER and elevations in serum CK activity. An undiagnosed gait abnormality, sore muscles and drop in energy level and unwillingness to perform after 5 -10 min of exercise are common complaints with type 2 PSSM. Warmbloods with type 2 PSSM have painful firm back and hindquarter muscles, reluctance to collect and engage the hindquarters, poor rounding over fences, gait abnormalities, and slow onset of atrophy especially when out of work. The mean age of onset of clinical signs in Warmbloods is between 8 and 11 years of age with the median CK and AST activity being 323 and 331U/L, respectively.

Diagnosis: Type 2 PSSM must be diagnosed by muscle biopsy where increased or abnormal PAS positive material that is usually amylase-sensitive is apparent particularly in subsarcolemmal locations. False positive diagnosis is possible for type 2 PSSM in highly trained horses that normally have higher muscle glycogen concentrations or in formalin fixed sections which show a greater deposition of subsarcolemmal glycogen even in healthy horses. Our laboratory grades polysaccharide accumulation as mild, moderate, and severe where mild accumulation represents a category which has a higher chance of being a false positive diagnosis. Mild PSSM cases in particular should receive a full physical examination to ensure that there are not other underlying causes for performance problems.

Management of PSSM

Signs of muscle pain, atrophy and stiffness in horses with both types of PSSM can be managed through specific diet and training regimes. Both diet and training must be changed to see a beneficial effect. The diet is altered to provide a moderate starch and sugar content, a slightly higher protein content with high quality amino acids and if needed for energy fat supplementation. This means reducing or replacing sweet feed, corn, wheat, oats, barley, and molasses with a ration balancer that contains vitamins, minerals and at least 20% protein. In addition, alfalfa hay, meal or cubes can be added to the diet. If the horse needs more energy to maintain weight, calories can be supplied in the form of fat. An equally important part of PSSM horse management is daily exercise. Consistent exercise enhances glycogen utilization, increases turnover of structural proteins in the muscle and builds enzymes needed to burn energy as fuel. At present, the best we can do is to use management techniques in order to reduce recurrence. Many horses with this muscle disorder have recurrent episodes of tying-up. An addition protein supplement such as Progressive's Topline extreme may be of value in horses with muscle atrophy and feeding after exercise may enhance its incorporation into muscle proteins. Ensure horses have normal serum vitamin E and supplement if necessary.

Avoid Rest: For chronic cases, prolonged rest after an episode appears to be counterproductive and predisposes PSSM horses to further episodes of muscle pain. With PSSM it is NOT advisable to only resume exercise when serum CK activity is normal. Rather, horses should begin small paddock turn out as soon as reluctance to move has abated. Providing daily turn out with compatible companions can be very beneficial as it enhances energy metabolism in PSSM horses. Grazing muzzles may be of benefit to PSSM horses turned out on pastures for periods when grass is particularly lush. Most PSSM horses are calm and not easily stressed, however, if stress is a precipitating fact, stressful environmental elements should be minimized.

Reintroducing exercise: Re-introduction of exercise after an acute episode of ER in PSSM horses needs to be gradual. Important principles include 1) providing adequate time for adaptation to a new diet before commencing exercise (2 weeks), 2) recognizing that the duration of exercise is more important to restrict than the intensity of exercise (no more than 5 min walk/trot to start) 3) ensuring that exercise is gradually introduced and consistently performed and 4) minimizing any days without some form of exercise. Exercise should begin with light slow uncollected work on a longe-line or under saddle beginning with once a day for 3-5 minutes at a walk and trot. This initial work should be very mild and very short in duration. Work at a walk and trot can be gradually increased by two minutes each day. When the horse can exercise for 15

minutes, a five-minute break at a walk can be provided, and then a few intervals of walk and trot can gradually be increased. At least three weeks of walk and trot should precede work at a canter.

Exercise: Regular daily exercise is extremely important for managing horses with PSSM. Even 10 min of exercise has been shown to be extremely beneficial in reducing muscle damage with exercise. Once conditioned, some PSSM horses thrive with 4 days of exercise as long as they receive daily turn out. For riding horses with type 2 PSSM, a prolonged warm-up with adequate stretching is recommended. Rest periods that allow horses to relax and stretch their muscles between 2 – 5 min periods of collection under saddle may be of benefit. Horses should be worked in a long and low frame for at least 4 weeks initially with a very gradual reintroduction of collected work. The collected work should be performed in intervals lasting no more than 5 min with a period of stretching provided between intervals. The time of active collection can be gradually increased as the horse works more underneath himself and in balance.

Try to exercise the horse on a daily basis even if only for 15 minutes on a lunge-line. Research has consistently shown that even 10-15 min a day will improve the function of muscle in PSSM horses. Once your horse is fit it may well manage with turn out rather than daily exercise from time to time. The number of days off a horse can manage is highly individual. If more than 3 or 4 days have gone by begin with a small amount of exercise.

A potential scenario for topline strength building exercises include: Relaxed work on a longeline before riding or just by itself 5 days a week. Aids that help create a long low frame may be useful such as Vienna reins. Begin with a couple of minutes of walk and then ask for a very relaxed trot to get the back swinging long and low without the need for hindlimb impulsion. Watch to see the base of his neck muscles release, the back stretch and rump muscles contract. To begin with, the length of time at a trot should be no more than 5 minutes in each direction. After that allow the horse to walk and stretch. After a few weeks, add canter after the trot is relaxed and continue foras long as the horse can carry himself with a relaxed base of his neck, impulsion and rounded back. Start with just a minute or less. When the horse releases at the base of its neck at the canter I would do a few more strides then come back to trot and do some trot with the horse moving well forward underneath him/herself and try to get him/her to stretch down at the same time. It takes several weeks in my experience to get them to round at a canter through their back. They often cannot hold the canter at first for more than a few strides in the correct position. Once this is working well add more transitions. After about 4 weeks If doing this for 20 minutes before you ride you should start see more energy and better carriage and with the diet change more muscle mass.

Dantrolene: For horses with highly recurrent forms of type 2 PSSM (Barrel horses for example), the use of dantrolene may be beneficial for a period of time in bringing horses back into training. A dose between 2 and 4 mg/kg should be given 1 hour before exercise and its peak effect is 90 min after administration. Dantrolene may cause a positive drug test in race horses or show horses. It should be gradually withdrawn once horses have settled into a training regime.

Dietary recommendations for PSSM

We strongly encourage owners to utilize an equine nutritionist to provide a balanced diet. Most feed companies have a nutritionist that will provide support for veterinarians. The Neuromuscular Diagnostic Laboratory does not have a nutritionist on staff but can work with a feed company to meet your horse's needs if you let us know what company you want to utilize.

Caloric balance: The first step in designing a diet for PSSM horses is to decide what the horse's caloric requirements are and what the horse's ideal body weight is or should be. Many horses with PSSM are easy keepers and may be overweight at the time of diagnosis. Adding excessive calories in the form of fat to an obese horse may produce metabolic syndrome and is contraindicated. If necessary, caloric intake can be

reduced by using a grazing muzzle during turn-out, feeding hay with a moderate nonstructural carbohydrate content (less than 12 % NSC) at 1 to 1.5% of body weight, providing a low calorie, protein supplemented ration balancer and gradually introducing daily exercise. Rather than provide dietary fat to an overweight horse, fasting for 6 h prior to exercise can be used to elevate plasma free fatty acids prior to exercise and alleviate any restrictions in energy metabolism in muscle.

Selection of forage: We recommend a high quality hay with an NSC content of about 12% NSC While it is important to feed a low NSC hay to horses with type 1 PSSM, this is likely not as important for type 2 PSSM. Our current recommendation for type 2 PSSM is to increase the quality of dietary protein by feeding half of the forage in the form of alfalfa hay or alfalfa cubes/meal/ and about half as grass hay. Of course this balance varies depending on the type of hay available in your area.

Low starch, fat enriched concentrates: A number of low starch, fat enriched concentrates are currently on the market and they have been tested on horses with type 1 PSSM. They may work very well for your horse with type 2 PSSM, however, the important principle to be met by such feeds is that they are fed in the amount recommended by the manufacturer on the feed bag or web site in order to provide the right balance of protein, vitamins and minerals. If that amount of feed produces excessive weight gain then select another product such as a ration balancer pellet with added protein (20 – 30%). Don't feed a lesser amount of the feed than recommended by the manufacturer, select a product better suited for your individual horse. Many times owners of horses with type 2 PSSM are so focused on low starch and high fat in the diet that they provide a diet that is deficient in the amino acids needed by the muscle and this exacerbated the horses condition.

Selection of fat source: If additional calories are needed to maintain weight, fat can be added to the diet. It may not be necessary for horses with type 2 PSSM to consume a high fat diet. We recommend providing some fat in the form of fish oil or flax seed, which has a high Omega 3 content. If more fat is needed to maintain weight and energy, oils or solid fats can be added. Suitable forms of vegetable oils include soybean, corn, safflower, canola, flaxseed, linseed, peanut and coconut or solid fat products may also be used. The amount of oil added to the diet varies for each horse and should not be provided in amounts that produce excessive weight gain or a cresty neck. Two cups of oil is often the maximum amount recommended. Due to the potential additional oxidant stress of fats, vitamin E (1000 – 6000 U/day) should be fed to horses receiving high oil diets.

Protein supplements: Addition of a protein supplement is recommended for type 2 PSSM horses that do not have an appropriate muscle mass, or horses that do not have the endurance/strength they need for higher level performance. Whey based formulations are recommended at the manufacturers recommended amount. Progressive's topline Xtreme or Purina's Supersport are two such supplements. Feeding within 45 min of exercise may enhance building muscle mass. If horses have evidence of a cresty neck, excessive fat pads or a history of metabolic syndrome or laminitis, sore feet consult your veterinarian before feeding higher protein feeds.

Expectations: It is important to note that a horse diagnosed with PSSM will always have an underlying predilection for muscle soreness and the best that can be done is to manage horses to minimize clinical signs. With adherence to both the diet and exercise recommendations about 70% of Warmblood horses show notable improvement in clinical signs and many return to acceptable levels of performance [14]. There is, however, a wide range in the severity of clinical signs shown by horses with PSSM; those horses with severe or recurrent clinical signs will require more stringent adherence to diet and exercise recommendations in order to regain muscle function.

Prognosis for PSSM

The best indicator as to whether horses with PSSM will be productive athletes is their past performance combined with response to changes in exercise regimes and diet. Genotype or biopsy findings cannot predict future athletic potential. Horses with PSSM will always have a predisposition to muscle soreness and will require longterm management of their condition.

Conflict of Interest Statement: Drs. Valberg, Mickelson and McCue own the license for PSSM testing and receive sales income from its use. Their financial and business interests have been reviewed and managed by the University in accordance with its conflict of interest policies. Dr. Valberg receives a portion of the profits of the sale of Re-Leve.
