# Supplementary material for: Muscle glycogen concentrations and response to diet and exercise regimes in Warmblood horses with type 2 Polysaccharide Storage Myopathy
Source: PLoS One. 2018 Sep 5;13(9):e0203467. doi: 10.1371/journal.pone.0203467 (PMC6124783; doi:10.1371/journal.pone.0203467)
Supplement: S3 File — Questionnaire distributed to participants of PSSM2 WB. (PDF) [file pone.0203467.s003.pdf]

Neuromuscular Diagnostic Lab: Muscle Biopsy Follow-up Survey

Thank you for your interest in Polysaccharide Storage Myopathies. We are constantly working to improve our recommendations for horses suffering from PSSM. We really need your feedback. Please take 15 -30 minutes to let us know how your horse is doing and what did and did not work. We will post the results from this survey, without any identifying or owner information, on our website once they have been analyzed and published in scientific journals.

For more information about the Equine Neuromuscular Diagnostic Lab at the Michigan State University's College of Veterinary Medicine, please visit:
http://cvm.msu.edu/research/faculty-research/valberg-laboratory

* Required

1. **Consent to Participate** *
   I understand that I am voluntarily completing this survey and allow my responses to be used in ongoing research with all identifying information removed. I understand that I may quit this survey at any time without penalty.
   Mark only one oval.

   - ( ) I consent to participate in this survey
   - ( ) I do not wish to participate in this survey. *Stop filling out this form.*

Thank you for your consideration of this survey. If you have additional questions, comments or concerns about this survey, the Neuromuscular Diagnostic Lab may be reached at nmdl@cvm.msu.edu

Basic Information

2. **Your Name:** *

   ____

3. **Phone number:**

   ____

4. **Email:** *

   ____
   ____
   ____
   ____
   ____

5. **Primary Veterinarian:**

   ____

6. **Primary Veterinarian's phone number:**

____

7. **Was this the same veterinarian that submitted the muscle biopsy?**
Check all that apply.

- [ ] Yes
- [ ] No
- [ ] Not sure

8. **Horse's name:** *

____

9. **Horse's year of birth:** *

____

10. **Gender:** *
Mark only one oval.

- ( ) Stallion
- ( ) Gelding
- ( ) Mare
- ( ) Ovariectomized mare

11. **Breed:** *

____

12. **Sire:**

____

13. **Dam:**

____

14. **Is this horse registered?**
Mark only one oval.

- ( ) Yes
- ( ) No
- ( ) Not sure

15. **If horse is registered, what breed registry(ies)?**

____

16. **If horse is registered, registered name:**

____

17. **How would you characterize your relationship to this horse?** *

Please check all that apply.
Check all that apply.

- [ ] Owner
- [ ] Primary rider
- [ ] Previous Owner
- [ ] Caretaker, but not rider or owner
- [ ] Other: ________________________

18. **How long have/had you owned or been associated with this horse?**

Mark only one oval.

- ◯ Less than 6 months
- ◯ 6 months- 1 year
- ◯ 2-4 years
- ◯ Greater than 5 years

19. **Do you still own/work with this horse?**

Mark only one oval.

- ◯ Yes
- ◯ No

20. **If no, how long after the muscle biopsy did this horse leave your care?**

(i.e. moved, sold or re-homed)
Mark only one oval.

- ◯ Less than 1 month
- ◯ 1-2 Months
- ◯ 3-6 months
- ◯ 7-11 months
- ◯ 1-2 years
- ◯ Greater than 2 years
- ◯ Other: ________________________

21. **Is this horse still living?**

Mark only one oval.

- ◯ Yes
- ◯ No
- ◯ Unsure

22. **If no, please indicate cause of death:**

....

....

....

....

....

Performance History

If horse is deceased or has been re-homed, please answer the following questions to the best of your ability. Please consider the months up until death or re-homing.

23. **What is/are the principle discipline(s) this horse is currently used for?**

Please check all that apply.
Check all that apply.

- [ ] Dressage
- [ ] Hunter/Jumper
- [ ] Eventing
- [ ] Driving
- [ ] Pleasure/recreation/trail
- [ ] Western pleasure
- [ ] Halter
- [ ] Racing
- [ ] Reining
- [ ] Cutting/sorting
- [ ] Endurance/competitive trail riding
- [ ] Breeding
- [ ] Not ridden
- [ ] Other: ....

24. **Please describe the horse's highest achieved level of training in the discipline(s) selected above and which year it was achieved in:**

....

....

....

....

....

Clinical Signs

Please describe the clinical signs that led you to submit a muscle biopsy.

25. **Did your horse exhibit any of the following signs? (check all that apply)**

Please check all that apply.
Check all that apply.

- [ ] Generalized muscle loss/atrophy
- [ ] Poor topline muscle
- [ ] One focal area of muscle loss/ atrophy
- [ ] Decline in performance
- [ ] Mild lameness
- [ ] Overall change in behavior
- [ ] Reluctance to go forward
- [ ] Reluctance to collect
- [ ] Difficulty with canter transitions or lead changes
- [ ] Difficulty backing up
- [ ] Bucking
- [ ] Tying up (Rhabdomyolysis- muscle stiffness, shortened hind limb stride, sweating, reluctance to move, very painful & sore croup muscle that are severely cramped)
- [ ] Muscle twitching/fasciculations
- [ ] Prolonged recumbency (lying down)
- [ ] Sensitivity to grooming
- [ ] Resentment of saddling or girthing
- [ ] Other: ....

26. **If your horse has episodes of "tying up" (rhabdomyolysis), when do they happen?**

Mark only one oval.

- ( ) With exercise
- ( ) Without exercise
- ( ) Both with and without exercise
- ( ) My horse has never "tied up"

27. **Prior to onset of clinical signs, did any of the following occur:**

Please check all that apply.
Check all that apply.

- [ ] Increase in exercise level
- [ ] Decrease in exercise level
- [ ] Increase in turnout time
- [ ] Decrease in turnout time
- [ ] Change in diet
- [ ] Significant medical event requiring veterinary attention
- [ ] Change in living situation (e.g. moved to new barn)
- [ ] Non of the above occured
- [ ] Other: ....

28. **How long did the clinical signs take to appear?**

Mark only one oval.

- So gradual I could not tell (3 or more months)
- Gradually (1-2 months)
- Suddenly (within a few days or weeks)
- Not sure
- Other: ____________________

29. **How old was this horse when clinical signs first occurred?**

Mark only one oval.

- Less than a year
- 1 year
- 2 years
- 3 years
- 4 years
- 5 years
- 6 years
- 7 years
- 8 years
- 9 years
- 10 years
- 11 years
- 12 years
- 13 years
- 14 years
- 15 years
- 16 years
- 17 years
- 18 years
- 19 years
- Greater that 20 years of age
- Not sure

30. **Was this horse in work during the time the muscle biopsy was taken?**

Mark only one oval.

- Yes
- No

31. **What percent decline in your horse's performance did you observe since the onset of clinical signs?**

Mark only one oval.

- ◯ 1-25%
- ◯ 26-50%
- ◯ 51-75%
- ◯ 76-99%
- ◯ 100%
- ◯ My horse did not decline in performance, but training did not advance as expected.

32. **After your horse's decline in performance, did you feel your horse would ever resume their normal activity or the potential to achieve your desired goals?**

Mark only one oval.

- ◯ Yes
- ◯ No
- ◯ Unsure

33. **Do you feel clinical signs were/are worse in the following weather conditions?**

Please check all that apply.

Check all that apply.

- ☐ Temperatures below freezing (0 degrees C or 32 degrees F)
- ☐ Cold weather
- ☐ Rain or wet weather
- ☐ Hot weather with low humidity
- ☐ Hot weather with high humidity
- ☐ No change with weather
- ☐ Other: ____________

34. **How would you rate the clinical signs after the horse has been off work or rested for a period of time?**

Mark only one oval.

- ◯ Better
- ◯ Worse
- ◯ No change
- ◯ Horse was always kept in work

35. **Please add any other comments on the clinical signs your horse displayed that led to a muscle biopsy.**

....

....

....

....

....

36. **Did you elect to do the PSSM type 1 genetic testing?**
Mark only one oval.

- ⬭ Yes
- ⬭ No

37. **If so what was the result?**
Mark only one oval.

- ⬭ N/N
- ⬭ P/N
- ⬭ P/P
- ⬭ Unsure

38. **From the muscle biopsy, what was this horse's diagnosis?**
Check all that apply.

- ☐ Type 1 PSSM
- ☐ Type 2 PSSM
- ☐ Myofibrillar Myopathy
- ☐ Unsure of diagnosis
- ☐ Other: ....

39. **In the 6 months prior to muscle biopsy, was a lameness detected?**
Mark only one oval.

- ⬭ Yes
- ⬭ No *Skip to question 53.*

Lameness History

40. **If a lameness was detected, did a veterinarian perform a lameness exam?**
Mark only one oval.

- ⬭ Yes
- ⬭ No

41. **If a lameness exam was performed, did the veterinarian feel they were able to isolate the cause of the lameness?**

Mark only one oval.

- Yes
- No

42. **Which leg(s) were affected by the lameness issues:**

Please check all that apply.
Check all that apply.

- Forelimbs
- Hindlimbs
- Unable to localize
- Other: ____________

43. **Which structures did a veterinarian diagnose as contributing to the lameness?**

Check all that apply.

- Cervical/ thoracic / lumbar spine
- Sacroiliac joint (SI)
- Hip
- Stifle
- Hock
- Feltlock
- Pastern
- Shoulder
- Elbow
- Knee
- Navicular
- Coffin
- Flexor Tendons
- Suspensory ligament
- Other: ____________

44. **What was the diagnosis provided by the veterinarian for the lameness:**

____

____

____

____

____

45. **Has your horse ever had a bone scan?**
Mark only one oval.

- ⬭ Yes
- ⬭ No

46. **What was the result of the bone scan?**
Mark only one oval.

- ⬭ Unknown
- ⬭ Positive for inflammation
- ⬭ Negative
- ⬭ This horse has never had a bone scan.

47. **If the bone scan was positive, which area had inflammation?**
Check all that apply.

- ☐ Forelimb
- ☐ Hindlimb
- ☐ Neck
- ☐ Back
- ☐ Hips or pelvis
- ☐ Don't know
- ☐ Other: ____________

48. **Was veterinary treatment for the lameness undertaken? (e.g. injections, stall rest, etc.)**
Mark only one oval.

- ⬭ Yes
- ⬭ No
- ⬭ Other: ____________

49. **If treatment for the lameness was undertaken, did you feel you had a satisfactory resolution?**
Mark only one oval.

- ⬭ Yes
- ⬭ No

50. **After the lameness was resolved did you feel the clinical signs had improved?**
Mark only one oval.

- ⬭ Yes
- ⬭ No
- ⬭ Lameness was not resolved
- ⬭ Other: ____________

51. **Does the lameness currently affect the level of this horse's performance?**
Mark only one oval.

- Yes
- No

52. **If yes, how does it affect performance?**

Exercise

Please answer the questions below pertaining to the current management of your horse with PSSM. If horse is deceased or has been re-homed, please answer the following questions to the best of your ability. Please consider the months up until death or re-homing.

53. **On average, how often does this horse currently get turnout?**
If this horse is currently on stall rest, please also check this horse's normal turn out schedule.
Check all that apply.

- [ ] No turnout
- [ ] Temporarily does not get turnout due to a veterinary issue (on stall rest)
- [ ] Less than 1 hour/ day
- [ ] 1-4 hours/day
- [ ] 5-9 hours/day
- [ ] More than 9 hours/day but still stalled daily
- [ ] Full pasture turnout

54. **On average, how often does this horse currently get worked under saddle or in harness?**
Mark only one oval.

- Not currently in work
- 1 time per week
- 2 times per week
- 3 times per week
- 4 times per week
- 5 times per week
- 6 times per week
- 7 times per week
- More than 7 times per week

55. **Approximately, how long does a typical ride under saddle last?**

Mark only one oval.

- Less than 20 min.
- 30 min.
- 45 min.
- 1 hour
- More than 1 hour
- Other: ____________

56. **Has your saddle been evaluated for your horse by a veterinarian or professional saddle fitter?**

Mark only one oval.

- Yes, my saddle fits.
- Yes, my saddle did not fit but I was able to get a saddle that does.
- Yes, my saddle does not fit.
- No

57. **How long do you warm up your horse before beginning to school or train them?**

Mark only one oval.

- Less than 5 minutes
- 6-10 minutes
- 11-15 minutes
- 16-20 minutes
- More than 20 minutes

58. **Please check all that apply to your warm up:**

Please note, long and low pertains to making/allowing the horse to stretch throughout their topline in a low frame or headset.

Check all that apply.

- [ ] Long and low framed lunging with aides (i.e. neck stretcher, pessoa, vienna reins, etc.)
- [ ] Long and low framed lunging without aides
- [ ] Unmounted stretching
- [ ] Long and low stretching under saddle prior to exercise
- [ ] Extended walk on a loose rein
- [ ] Trotting in set time intervals
- [ ] Trotting on a loose rein
- [ ] Canter on a loose rein
- [ ] No specific warm up
- [ ] Other: ____________

59. **How many breaks do you, on average, provide your horse during your ride?**
Please consider a break to be a walk with no collection.
Mark only one oval.

- No breaks
- 1 break
- 2 breaks
- 3 breaks
- 4 breaks
- 5 or more breaks

60. **On average, how many days a week does your horse receive NO form of exercise?**
i.e. Stall rest with no turn out, no hand walking, no lunging, etc.
Mark only one oval.

- 0 days
- 1 day
- 2 days
- 3 days
- 4 days
- 5 days
- 6 days
- 7 days

61. **Did you change this horse's exercise regime after the biopsy results?**
Mark only one oval.

- Yes
- No *Skip to question 71.*
- Partially

Change in Exercise Regime

62. **Did you try the recommendation we made for warming up on a lunge line with the horse stretching the topline in a long and low frame?**
Mark only one oval.

- Yes, I felt it helped
- Yes, but I saw no difference
- No

63. **If you answered no to the previous question, please explain why:**

....

....

....

....

....

64. **Please mark the changes you made to this horse's exercise regime after the muscle biopsy diagnosis:**

Please check all that apply. Please note, long and low pertains to making/allowing the horse to stretch throughout their topline in a low frame or headset.

Check all that apply.

- [ ] Started warming up with long and low framed stretching on the lunge line
- [ ] Started warming up with long and low framed stretching under saddle
- [ ] Increased my warm up time
- [ ] Decreased my warm up time
- [ ] Increased the intensity of work within the same riding time
- [ ] Decreased the intensity of work within the same riding time
- [ ] Increased riding time with the same level of intensity
- [ ] Decreased riding time with the same level of intensity
- [ ] Increased both riding time and level of intensity
- [ ] Decreased both riding time and level of inensity
- [ ] Allow frequent breaks during riding
- [ ] Work my horse more often (more times per week)
- [ ] Give my horse more days off
- [ ] Changed disciplines
- [ ] Increased amount of turn out
- [ ] Decreased amount of turn out
- [ ] Other: ....

65. **Did you change disciplines after the muscle biopsy?**

Mark only one oval.

- ( ) Yes
- ( ) No

66. **If yes, what your horse's PREVIOUS discipline(s) before the muscle biopsy?**

Please check all that apply.

Check all that apply.

- [ ] Dressage
- [ ] Hunter
- [ ] Jumper
- [ ] Eventing
- [ ] Driving
- [ ] Pleasure/recreational/trail
- [ ] Western pleasure
- [ ] Reining
- [ ] Hatler
- [ ] Racing
- [ ] Cutting/sorting
- [ ] Endurance
- [ ] Breeding
- [ ] Not ridden

67. **What is your horse's CURRENT discipline?**

....

68. **Do you feel any change in your horse's exercise routine improved clinical signs?**

Mark only one oval.

- ( ) Yes
- ( ) No
- ( ) Cannot tell

69. **How long after you changed your exercise routine did you notice any improvement?**

Mark only one oval.

- ( ) Less than 1 month
- ( ) 1-2 Months
- ( ) 3-4 months
- ( ) Greater than 4 months

70. **Please describe the exercise change you felt yielded the most improvement:**

....

....

....

....

....

Health History

Please answer the health questions below and rate your horse's body condition score using the pictures and descriptions. If horse is deceased or has been re-homed, please answer the following questions to the best of your ability. Please consider the months up until death or re-homing.

71. **Has your horse ever been diagnosed with Cushing's disease (PPID)?**

Mark only one oval.

- Yes
- No
- Not sure

72. **Has this horse ever been diagnosed by a veterinarian to have the following:**

Please check all that apply.

Check all that apply.

- [ ] Shivers
- [ ] Stringhalt
- [ ] EPM
- [ ] Not sure
- [ ] None of the Above

73. **Has your horse ever been diagnosed with laminitis?**

Mark only one oval.

- Yes
- No
- Not sure

74. **Has your horse ever been diagnosed with colic?**

Mark only one oval.

- Yes
- No
- Not sure

75. **Has your horse ever been diagnosed with respiratory disease such as heaves?**

Mark only one oval.

- Yes
- No
- Not sure

76. **Has this horse ever been diagnosed by a veterinarian to have gastrointestinal ulcers?**
Mark only one oval.

- ( ) Yes, via endoscopy
- ( ) Yes, only by exhibited symptoms
- ( ) No
- ( ) Not sure

77. **If yes, what kind of ulcers?**
Mark only one oval.

- ( ) Gastric (stomach)
- ( ) Hind gut
- ( ) Other: ____________

78. **If so, how was this horse treated for ulcers?**
Check all that apply.

- [ ] Gastroguard
- [ ] Ulcerguard
- [ ] Sucralfate
- [ ] Ranitidine
- [ ] Succeed hind gut supplement
- [ ] Other hind gut buffer
- [ ] Other: ____________

79. **Did the clinical signs that led to a muscle biopsy show any improvement with the treatment of gastric ulcers?**
Mark only one oval.

- ( ) Yes
- ( ) No
- ( ) Unsure
- ( ) Ulcers did not occur at the same time as the clinical signs that led to a muscle biopsy

80. **Has your horse had vitamin E levels measured by your veterinarian?**
Mark only one oval.

- ( ) Yes
- ( ) No
- ( ) Not sure

81. **If yes, what was your horse's vitamin E level**
Mark only one oval.

- Deficient (<1.5 ug/ml)
- Marginal (1.5-2 ug/ml)
- Adequate (>2 ug/ml)
- I'm not sure/can't remember

82. **How would you evaluate your horse's body condition score (BCS) BEFORE the muscle biopsy?**
Consider 4-5 an ideal body weight. Please use the pictures and descriptions below for reference.
Mark only one oval.

	1	2	3	4	5	6	7	8	9	
extremely thin										obese

Body Condition Score Chart

Areas of emphasis for body condition scoring: thickening of the neck, fat covering the withers, fat deposits along backbone, fat deposits on flanks, fat deposits on inner thighs, fat deposits around tailhead, fat deposits behind shoulders, fat covering ribs, shoulder blends into neck

Kentucky Equine Research®

859-873-1988, www.ker.com

1 Poor

Animal extremely emaciated; spine, ribs, tailhead, points of hip and buttock projecting prominently; bone structure of withers, shoulders, and neck easily noticeable; no fatty tissue can be felt.

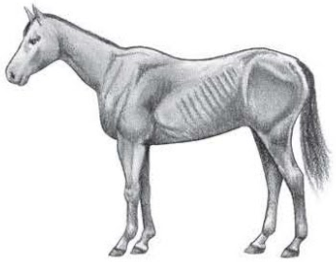

2 Very Thin

Animal emaciated; slight fat covering over base of spine; ribs, tailhead, points of hip and buttock prominent; withers, shoulders, and neck structure faintly discernable.

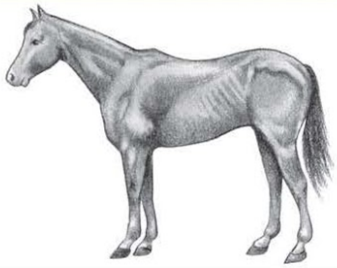

3 Thin

Fat buildup about halfway on spine; slight fat cover over ribs; spine and ribs easily discernable; tailhead prominent, but individual vertebrae cannot be identified visually; points of hip appear rounded but easily discernable; points of buttock not distinguishable; withers, shoulders, and neck accentuated.

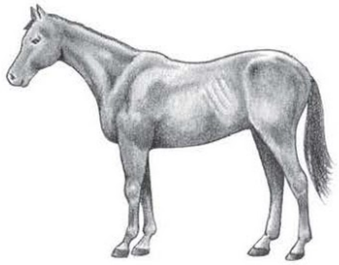

4 Moderately Thin

Slight ridge along back; faint outline of ribs discernable; tailhead prominence depends on conformation, fat can be felt around it; points of hip not discernable; withers, shoulders, and neck not obviously thin.

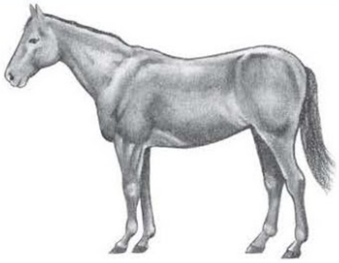

5 Moderate

Back is flat (no crease or ridge); ribs not visually distinguishable but easily felt; fat around tailhead beginning to feel spongy; withers appear rounded over spine; shoulders and neck blend smoothly into body.

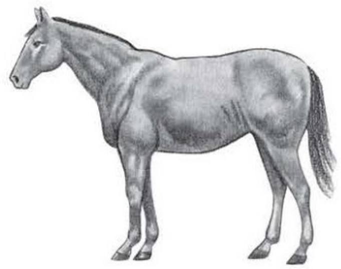

6 Moderately Fleshy

May have slight crease down back; fat over ribs fleshy/spongy; fat around tailhead soft; fat beginning to be deposited along sides of withers, behind shoulders, and along sides of neck.

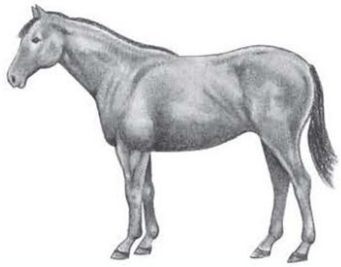

7 Fleshy

May have crease down back; individual ribs can be felt, but noticeable filling between ribs with fat; fat around tailhead soft; fat deposited along withers, behind shoulders, and along neck.

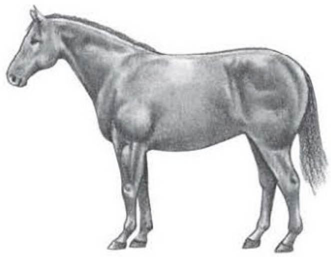

8 Fat

Crease down back; difficult to feel ribs; fat around tailhead very soft; area along withers filled with fat; area behind shoulders filled with fat; noticeable thickening of neck; fat deposited along inner thighs.

9 Extremely Fat

Obvious crease down back; patchy fat appearing.

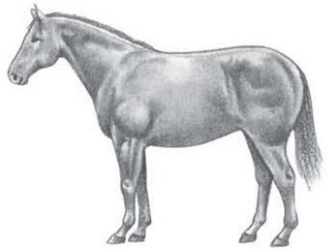

Kentucky Equine Research, 3910 Delaney Ferry Rd., Versailles, KY 40383, 859-873-1988, www.ker.com

© Copyright Kentucky Equine Research, 2010

83. **How would you evaluate your horse's body condition score (BCS) AFTER changes made from the muscle biopsy results?**

Consider 4-5 an ideal body weight. Please use the pictures and descriptions above for reference.
Mark only one oval.

	1	2	3	4	5	6	7	8	9	
extremely thin	◯	◯	◯	◯	◯	◯	◯	◯	◯	obese

84. **How would you rate your horse's appetite?**

Consider a rating of 3 as average.

Mark only one oval.

	1	2	3	4	5	
Poor	◯	◯	◯	◯	◯	Superb

85. **Do/did you consider your horse an easy keeper?**

Mark only one oval.

- ◯ Yes, he/she is an easy keeper
- ◯ No, my horse maintains weight with the appropriate amount of food
- ◯ No, my horse is VERY hard to keep weight on

86. **Does/did your horse have a "cresty" neck?**

Mark only one oval.

- ◯ Yes
- ◯ No
- ◯ Not sure

87. **Has/had your horse ever been diagnosed with Equine Metabolic Syndrome or insulin resistance?**

Mark only one oval.

- ◯ Yes
- ◯ No
- ◯ Not Sure

88. **Has this horse ever been bred?**

Mark only one oval.

- ◯ Yes
- ◯ No
- ◯ Not sure

Previous Diet- BEFORE Biopsy

Please answer the following questions about your horse's diet BEFORE a biopsy or test yielding a diagnosis was made.

89. **Please check the components of your horse's previous diet (before the muscle biopsy):**
Please check all that apply.
Check all that apply.

- [ ] Fresh grass (i.e. pasture)
- [ ] Dried forage (i.e. hay)
- [ ] Packaged forage (i.e. hay cubes)
- [ ] Complete feed with no forage (i.e. equine senior type feed)
- [ ] Concentrates (i.e. most maintenance grains, sweet-feed, etc.)
- [ ] Low starch, high fat concentrate (i.e. Releve, Ultium, rice bran, etc.)
- [ ] Low starch ration Balancer
- [ ] Fat supplement (i.e. oil, cool calories)
- [ ] Vitamin or mineral supplement
- [ ] Other: ....

90. **What type of hay was fed?**
Please check all that apply.
Check all that apply.

- [ ] Alfalfa
- [ ] Grass Hay (Orchard, Bermuda, Timothy, etc)
- [ ] Grass & Alfalfa Mix
- [ ] I'm not sure
- [ ] No hay was fed
- [ ] Other: ....

91. **How often did your horse have access to fresh grass?**
Mark only one oval.

- ( ) 1-3 months per year
- ( ) 4-6 months per year
- ( ) 6-8 months per year
- ( ) More than 8 months per year
- ( ) Never, fresh forage in unavailable

92. **If you fed grain or a complete feed, what product(s) did you feed?**
Please check all that apply.
Check all that apply.

- [ ] Grain concentrate (i.e. Maintenance Adult, Maintenance Jr, oats, sweet-feed, etc.)
- [ ] Complete senior feed (i.e. Purina Equine Senior, etc.)
- [ ] Low starch feed containing fat (i.e. Releve, Ultium, rice bran)
- [ ] Low starch ration balancer
- [ ] Other: ....

93. **If you supplemented your horse's diet, what did you supplement with?**
Mark only one oval.

- Fat supplement (i.e. Oil, Cool Calories, Envision, Amplify, etc.)
- Protein supplement (i.e. Progressive's Topline Xtreme, Purina Supersport, ration balancer with added protein (20-30%)
- ALCAR or Acylcarnitine
- Magnesium
- Vitamin E
- Broad spectrum vitamin/mineral supplement
- Other: ____________________

94. **Following the results of the muscle biopsy, did you change your horse's diet?**
Mark only one oval.

- Yes
- No, my horse is on the same diet as before (described above) *Skip to question 103.*

DIET AFTER DIAGNOSIS

Please use this section to describe to changes you made to this horse's diet AFTER the muscle biopsy or test yielding a diagnosis was made. If horse is deceased or has been re-homed, please answer the following questions to the best of your ability. Please consider the months up until death or re-homing.

95. **Did you follow the recommended dietary changes?**
Mark only one oval.

- Yes
- No
- Partly
- Other: ____________________

96. **If no, please explain why:**

____

____

____

____

____

97. **Please indicate all the changes you made:**
Please check all that apply.
Check all that apply.

- [ ] Changed source of calories (carbohydrates and fats)
- [ ] Changed protein intake (alfalfa hay and protein supplements)
- [ ] Change in supplementation (ration balancers, multivitamins, vitamin E, etc.)
- [ ] Changed access to fresh grass or grazing time
- [ ] Changed medication
- [ ] Change in feeding frequency
- [ ] Change in amount of feed
- [ ] Change in feeding times
- [ ] Other: ....

98. **How did you change your horse's source of calories?**
Please check all that apply.
Check all that apply.

- [ ] Decreased the portion of grass hay fed
- [ ] Increased the portion of grass hay fed
- [ ] Decreased the portion of "other" hay fed (not alfalfa or grass)
- [ ] Increased the portion of "other" hay fed (not alfalfa or grass)
- [ ] Decreased the portion of alfalfa hay fed
- [ ] Increased the portion of alfalfa hay fed
- [ ] Decreased portion of fat supplement (oil, cool calories, etc.) fed
- [ ] Added or increased a fat supplement (oil, cool calories, etc.) fed
- [ ] Decreased portion of complete feed of grain concentrate
- [ ] Increased portion of complete feed of grain concentrate
- [ ] Decreased amount of low starch, high fat concentrate (Releve, Ultium, rice bran)
- [ ] Increased amount of low starch, high fat concentrate (Releve, Ultium, rice bran)
- [ ] Decreased low starch ration balancer
- [ ] Added or increased a low starch ration balancer
- [ ] Decreased grazing time on fresh grass
- [ ] Increased grazing time on fresh grass
- [ ] Muzzled horse during turn out
- [ ] Other: ....

99. **What source of fat supplementation do you feed?**
Please check all that apply.
Check all that apply.

- [ ] I do not feed a fat supplementation
- [ ] Low starch feed with fat (i.e. rice bran)
- [ ] Solid source of fat (i.e. Cool Calories)
- [ ] Fish oil (high in Omega 3)
- [ ] Flax seed oil (high in Omega 3)
- [ ] Soybean oil
- [ ] Corn oil
- [ ] Safflower oil
- [ ] Canola oil
- [ ] Linseed oil
- [ ] Peanut oil
- [ ] Coconut oil
- [ ] Other: ____________________

100. **What source of protein do you feed?**
Please check all that apply.
Check all that apply.

- [ ] I do not feed a protein source
- [ ] Alfalfa hay
- [ ] Progressive's Topline Xtreme
- [ ] Purina's Supersport
- [ ] Ration balancer with added protein (20-30%)
- [ ] Other: ____________________

101. **Do you supplement your horse's current feed with any of the following?**
Mark only one oval.

- ( ) Vitamin E (1000-6000 IU per day)
- ( ) Magnesium
- ( ) Broad spectrum vitamin/mineral supplement
- ( ) ALCAR or Acylcarnitine
- ( ) Other: ____________________

102. **Do you feel the change in diet helped improve clinical signs?**
Mark only one oval.

- ( ) Yes
- ( ) No
- ( ) Cannot tell

FOLLOW-UP CARE

If horse is deceased or has been re-homed, please answer the following questions to the best of your ability. Please consider the months up until death or re-homing.

103. **What year did your horse receive a diagnosis via muscle biopsy?**

Mark only one oval.

- 2006
- 2007
- 2008
- 2009
- 2010
- 2011
- 2012
- 2013
- 2014
- 2015
- 2016

104. **Overall, have you noticed any improvement in the initial clinical signs?**

Mark only one oval.

- Yes
- No
- Not sure

105. **What percent IMPROVEMENT have you noticed in your horse's performance since altering their management?**

Mark only one oval.

- I have observed no improvement (0%)
- 1-25%
- 26-50%
- 51-75%
- 76-99%
- 100%

106. **Please indicate any improvements seen:**
Please check all that apply.
Check all that apply.

- [ ] Improved muscle mass
- [ ] Has resumed training or performance level
- [ ] Improved gait or lameness
- [ ] Willingly maintains forward motion
- [ ] Collects when asked
- [ ] Improved canter transitions
- [ ] Has no problem backing up
- [ ] Less or no bucking
- [ ] No longer ties-up
- [ ] No noted muscle twitches/ fasiculations
- [ ] Behaves as normal self
- [ ] Less or no resistance to saddling or girthing
- [ ] Less or no objection to grooming
- [ ] Other: ____________

107. **What percent DECLINE in your horse's performance have you observed since changing their management?**
Mark only one oval.

- ( ) I have observed no decline in performance(0%) and training is advancing as expected.
- ( ) I have observed no decline (0%), but training has not advanced as expected
- ( ) 1-25%
- ( ) 26-50%
- ( ) 51-75%
- ( ) 76-99%
- ( ) 100%

108. **Please list any additional details about treatment pursued following muscle biopsy results**
Including medication, integrative therapy, etc.

____
____
____
____
____

109. **Which clinical signs does this horse still exhibit?**
Please check all that apply.
Check all that apply.

- [ ] General muscle loss/ atrophy
- [ ] Poor topline muscle
- [ ] One focal area of muscle loss/ atrophy
- [ ] Decline in performance
- [ ] Mild lameness
- [ ] Reluctance to go forward
- [ ] Reluctance to collect
- [ ] Difficulty backing up
- [ ] Tying up (rhabdomyolysis)
- [ ] Muscle twitching/fasciculations
- [ ] Prolonged recumbency (lying down)
- [ ] Difficulty with canter transitions
- [ ] Bucking
- [ ] Overall change in behavior
- [ ] Sensitivity to grooming
- [ ] Resents saddling/ girthing
- [ ] Doesn't exhibit any clinical signs
- [ ] Other: ....

110. **If clinical signs still occur, please describe the severity and how often the clinical signs are noted:**
Example: Clinical sign is better but still present, clinical sign is worse, clinical sign has not changed...

....
....
....
....
....

Additional Information

111. **Please take this time to include any additional information you feel may be of importance to our study.**

....
....
....
....
....

Equine Neuromuscular Diagnostic Laboratory

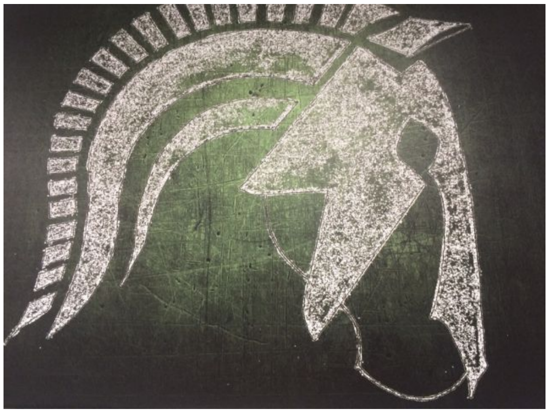

112. **How satisfied were you with the biopsy process?**

Including taking the biopsy, results and follow-up communication

Mark only one oval.

	1	2	3	4	5	6	7	8	9	10	
extremely dissatisfied	◯	◯	◯	◯	◯	◯	◯	◯	◯	◯	extremely satisfied

113. **Did your horse have any complications as a result of taking the biopsy?**

Mark only one oval.

◯ Yes

◯ No

114. **If yes, please describe:**

....

115. **Do you have any additional comments on the biopsy process?**

....

....

....

....

....

116. **How did you hear about the Equine Neuromuscular Diagnostic Lab?**
Mark only one oval.

- Referring Veterinarian
- Magazine Article
- Internet Publication
- Friend
- Website
- Other: ____________

117. **Were you satisfied with the time it took to receive the results?**
Mark only one oval.

- Yes
- No

118. **Were you satisfied with the treatment recommendations made with the diagnosis?**
Mark only one oval.

- Yes
- No
- Other: ____________

119. **If ongoing research provides additional information on your horse's case, would you like us to contact you?**
Due to client confidentiality, only owners and referring veterinarians will be contacted.
Mark only one oval.

- Yes
- No
