# Supplementary material for: Muscle glycogen concentrations and response to diet and exercise regimes in Warmblood horses with type 2 Polysaccharide Storage Myopathy
Source: PLoS One. 2018 Sep 5;13(9):e0203467. doi: 10.1371/journal.pone.0203467 (PMC6124783; doi:10.1371/journal.pone.0203467)
Supplement: S4 File — Questionnaire distributed to baseline WB participants. (PDF) [file pone.0203467.s004.pdf]

Equine Neuromuscular Diagnostic Lab: Healthy Performance Horse Questionnaire

The Equine Neuromuscular Diagnostic Lab is dedicated to providing the most accurate diagnosis and optimal treatment of muscle disorders in horses. Whenever we evaluate how our recommendations for affected horses are working, we need to compare them to the lives of healthy horses. We need responses from owners of healthy horses to figure out what impact management has on muscle disorders and to see if we are making a significant difference to their health and performance.

Please, for the betterment of the performance horse, answer this survey based on your healthy horse (defined as a horse currently being ridden and never known to have a muscle disease). It will take approximately 15 minutes and means so much for our research.

For more information about the Equine Neuromuscular Diagnostic Lab at the Michigan State University's College of Veterinary Medicine, please visit:
http://cvm.msu.edu/research/faculty-research/valberg-laboratory

* Required

1. **Consent to Participate** *

   I understand that I am voluntarily completing this survey and allow my responses to be used in ongoing research with all identifying information removed. I understand that I may quit this survey at any time without penalty.
   Mark only one oval.

   - ( ) I consent to participate in this survey
   - ( ) I do not wish to participate in this survey. *Stop filling out this form.*

Thank you for your consideration of this survey. If you have additional questions, comments or concerns about this survey, the Equine Neuromuscular Diagnostic Lab may be reached at nmdl@cvm.msu.edu

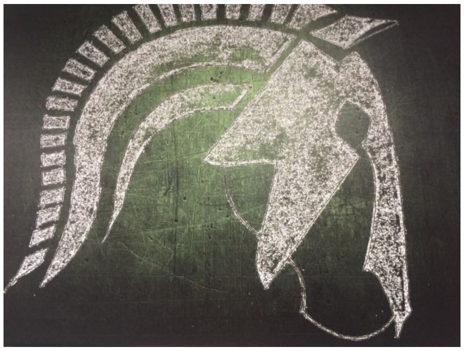

Basic Information

2. **Your Name:** *

   ____

3. **Phone number:**

   ____

4. **Email:** *

5. **Primary Veterinarian:**

6. **Primary Veterinarian's phone number:**

7. **Horse's name:** *

8. **Horse's year of Birth:** *
Mark only one oval.

- 1990
- 1991
- 1992
- 1993
- 1994
- 1995
- 1996
- 1997
- 1998
- 1999
- 2000
- 2001
- 2002
- 2003
- 2004
- 2005
- 2006
- 2007
- 2008
- 2009
- 2010
- 2011
- 2012
- 2013
- 2014
- 2015
- 2016

9. **Gender:** *
Mark only one oval.

- Stallion
- Gelding
- Mare
- Ovariectomized mare

10. **Breed:** *

....

11. **Sire:**

12. **Dam:**

13. **Is this horse registered?**
Mark only one oval.

- ( ) Yes
- ( ) No

14. **If horse is registered, what breed registry(ies)?**

15. **If horse is registered, registry name and number:**

16. **How would you characterize your relationship to this horse?** *
Please check all that apply.
Check all that apply.

- [ ] Owner
- [ ] Primary rider
- [ ] Previous owner
- [ ] Caretaker, but not rider or owner
- [ ] Other:

17. **How long have/had you owned or been associated with this horse?**
Mark only one oval.

- ( ) Less than 6 months
- ( ) 6 months- 1 year
- ( ) 2-4 years
- ( ) Greater than 5 years

Performance History

18. **What is/are the principle discipline(s) this horse is currently used for?**
Please check all that apply.
Check all that apply.

- [ ] Dressage
- [ ] Hunter/ Jumper
- [ ] Eventing
- [ ] Driving
- [ ] Pleasure/recreation/trail
- [ ] Western Pleasure
- [ ] Halter
- [ ] Reining
- [ ] Cutting/sorting
- [ ] Racing
- [ ] Endurance
- [ ] Breeding
- [ ] Not ridden
- [ ] Other: ....

19. **Please describe the horse's highest achieved level of training in the discipline(s) selected above and which year it was achieved in:**

....
....
....

....

20. **How often does this horse currently get turned out?**
If horse is currently on stall rest, please also indicate the normal amount of time this horse is turned out.
Check all that apply.

- [ ] No turn out
- [ ] Temporarily does not get turned out due to a veterinary issue (on stall rest)
- [ ] Less than 1 hour/day
- [ ] 1-4 hours/day
- [ ] 5-9 hours/day
- [ ] More than 9 hours/day but still stalled daily
- [ ] Full pasture turn out

21. **How often does this horse currently get worked under saddle?**
Mark only one oval.

- Not currently in work
- 1 time per week
- 2 times per week
- 3 times per week
- 4 times a week
- 5 times per week
- 6 times per week
- 7 or more times per week

22. **Approximately, how long does a typical ride/drive last?**
Mark only one oval.

- Less than 20 min.
- 30 min.
- 45 min.
- 1 hour
- More than 1 hour
- Other:

23. **Has your saddle been evaluated for your horse by a veterinarian or professional saddle fitter?**
Mark only one oval.

- Yes, my saddle fits.
- Yes, my saddle did not fit but I was able to get a saddle that does.
- Yes, my saddle does not fit.
- No

24. **How long do you warm up your horse before beginning to school or train them?**
Mark only one oval.

- Less than 5 minutes
- 6-10 minutes
- 11-15 minutes
- 16-20 minutes
- More than 20 minutes

25. **Please check all that apply to your warm up:**

Please note, long and low pertains to making/allowing the horse to stretch throughout their topline in a low frame or headset.

Check all that apply.

- [ ] Long and low lunging with aides (i.e. neck stretcher, pessoa, vienna reins, etc.)
- [ ] Long and low lunging without aides
- [ ] Unmounted stretching
- [ ] Long and low stretching under saddle prior to exercise
- [ ] Extended walk on a loose rein
- [ ] Trotting in set time intervals
- [ ] Trotting on a loose rein
- [ ] Canter on a loose rein
- [ ] No specific warm up
- [ ] Other: ....

26. **How many breaks do you, on average, provide your horse during your ride?**

Please consider a break to be a walk with no collection.

Mark only one oval.

- ( ) No breaks
- ( ) 1 break
- ( ) 2 breaks
- ( ) 3 breaks
- ( ) 4 breaks
- ( ) 5 or more breaks

27. **On Average, how many days a week does your horse receive NO form of exercise?**

i.e. Stays in their stall- no turnout, no lunging, no handwalking

Mark only one oval.

- ( ) 0 days
- ( ) 1 day
- ( ) 2 days
- ( ) 3 days
- ( ) 4 days
- ( ) 5 days
- ( ) 6 days
- ( ) 7 days

28. **Does your horse currently exhibit any of the following?**
Please check all that apply.
Check all that apply.

- [ ] Decline in performance
- [ ] Reluctance to collect
- [ ] Difficulty backing up
- [ ] Difficulty with canter transitions or lead changes
- [ ] Reluctance to go forward
- [ ] Bucking
- [ ] Overall Change in behavior
- [ ] Mild lameness
- [ ] Tying up (Rhabdomyolysis- muscle stiffness, shortened hind limb stride, sweating, reluctance to move, very painful & sore croup muscle that are severely cramped)
- [ ] Muscle twitching/ fasiculations
- [ ] Generalized muscle loss/ atrophy
- [ ] One focal area of muscle loss/ atrophy
- [ ] Poor topline muscle
- [ ] Prolonged recumbancy (lying down)
- [ ] Sensitivity to grooming
- [ ] Resentment of saddling or girthing

29. **If your horse has episodes of "tying up" (rhabdomyolysis), when do they happen?**
Mark only one oval.

- ( ) With exercise
- ( ) Without exercise
- ( ) Both with and without exercise
- ( ) My horse has never "tied up"

30. **In the past year, has your horse suffered from a lameness?**
Mark only one oval.

- ( ) Yes
- ( ) No *Skip to question 43.*

Lameness History

31. **If a lameness was detected, did a veterinarian perform a lameness exam?**
Mark only one oval.

- ( ) Yes
- ( ) No

32. **If a lameness exam was performed, did the veterinarian feel they were able to isolate the cause of the lameness?**

Mark only one oval.

- Yes
- No

33. **Which leg(s) were affected by the lameness issue(s):**

Please check all that apply.
Check all that apply.

- Forelimb
- Hindlimb
- Unable to localize which limb was lame

34. **Which structures did a veterinarian diagnose as contributing to the lameness?**

Please check all that apply.
Check all that apply.

- Cervical/ thoracic / lumbar spine
- Sacroiliac joint (SI)
- Hip
- Stifle
- Hock
- Fetlock
- Pastern
- Shoulder
- Elbow
- Knee
- Navicular
- Coffin
- Flexor tendons
- Suspensory ligament
- Other: ____________________

35. **What was the diagnosis provided by the veterinarian for the lameness:**

____

____

____

36. **Has your horse ever had a bone scan?**

Mark only one oval.

- ( ) Yes
- ( ) No

37. **What was the result of the bone scan?**

Mark only one oval.

- ( ) Unknown
- ( ) Positive for inflammation
- ( ) Negative

38. **If the bone scan was positive, which area had inflammation?**

Please check all that apply.
Check all that apply.

- [ ] Neck
- [ ] Back
- [ ] Hips or pelvis
- [ ] Forelimb
- [ ] Hindlimb
- [ ] Don't know
- [ ] Other: ....

39. **Was veterinary treatment for the lameness undertaken? (e.g. injections, stall rest, etc.)**

Mark only one oval.

- ( ) Yes
- ( ) No
- ( ) Other: ....

40. **If treatment for the lameness was undertaken, did you feel you had a satisfactory resolution?**

Mark only one oval.

- ( ) Yes
- ( ) No

41. **Does the lameness currently affect the level of this horse's performance?**

Mark only one oval.

- ( ) Yes
- ( ) No

42. **If yes, how does it affect performance?**

Diet

43. **Please check to components of your horse's feed:**
Please check all that apply.
Check all that apply.

- [ ] Fresh grass (i.e. pasture)
- [ ] Dried forage (i.e. hay)
- [ ] Packaged forage (i.e. hay cubes)
- [ ] Complete feed with no forage (i.e equine senior type feed)
- [ ] Concentrates (i.e. most grains)
- [ ] Low starch, high fat concentrate (i.e. Releve, Ultium, rice bran)
- [ ] Low starch ration Balancer
- [ ] Fat supplement (i.e. oil, cool calories)
- [ ] Vitamin or mineral supplement
- [ ] Other:

44. **What type of hay do you feed?**
Please check all that apply.
Check all that apply.

- [ ] Alfalfa
- [ ] Grass hay (Orchard, Bermuda, Timothy, etc.)
- [ ] I am not sure
- [ ] No hay is fed
- [ ] Other:

45. **How often does your horse have access to fresh grass?**
Mark only one oval.

- ( ) 1-3 months per year
- ( ) 4-6 months per year
- ( ) 6-8 months per year
- ( ) More than 8 months per year
- ( ) Never, fresh forage in unavailable

46. **If you feed grain or a complete feed, what product(s) do you feed?**

Please check all that apply.
Check all that apply.

- [ ] Grain concentrate (i.e. Maintenance Adult, Maintenance Jr, oats, sweetfeed, etc.)
- [ ] Complete senior feed (i.e. Purina Equine Senior, etc.)
- [ ] Low starch feed containing fat (i.e. rice bran)
- [ ] Low starch ration balancer
- [ ] Other: ....

47. **What source of fat supplementation do you feed?**

Please check all that apply.
Check all that apply.

- [ ] I do not feed a fat supplementation
- [ ] Low starch feed with fat (i.e. rice bran)
- [ ] Solid source of fat (i.e. Cool Calories)
- [ ] Fish oil (high in Omega 3)
- [ ] Flax seed oil (high in Omega 3)
- [ ] Soybean oil
- [ ] Corn oil
- [ ] Safflower oil
- [ ] Canola oil
- [ ] Linseed oil
- [ ] Peanut oil
- [ ] Coconut oil
- [ ] Other: ....

48. **What source of protein do you feed?**

Please check all that apply.
Check all that apply.

- [ ] I do not feed a protein source
- [ ] Alfalfa hay
- [ ] Progressive's Topline Xtreme
- [ ] Purina's Supersport
- [ ] Ration balancer with added protein (20-30%)
- [ ] Other:

49. **If you supplement your horse's diet, what do you supplement with?**

Please check all that apply.

Check all that apply.

- [ ] Vitamin E (1000-6000 IU per day)
- [ ] Magnesium
- [ ] Broad spectrum vitamin/mineral supplement
- [ ] ALCAR or Acylcarnitine
- [ ] Other: ____________________

Health History

Please answer the health questions below and rate your horse's body condition score using the pictures and descriptions.

50. **Has this horse ever been diagnosed with Cushing's disease (PPID)?**

Mark only one oval.

- ( ) Yes
- ( ) No
- ( ) Not sure

51. **Has this horse ever been diagnosed by a veterinarian to have the following:**

Check all that apply

Check all that apply.

- [ ] Shivers
- [ ] Stringhalt
- [ ] EPM
- [ ] Not sure
- [ ] None of the above

52. **Has this horse ever been diagnosed with laminitis?**

Mark only one oval.

- ( ) Yes
- ( ) No
- ( ) Not sure

53. **Has this horse ever been diagnosed with colic?**

Mark only one oval.

- ( ) Yes
- ( ) No
- ( ) Not Sure

54. **Has this horse ever been diagnosed with respiratory disease such as heaves?**

Mark only one oval.

- Yes
- No
- Not sure

55. **Has this horse ever been diagnosed by a veterinarian to have gastrointestinal ulcers?**

Mark only one oval.

- Yes, via endoscopy
- Yes, only by exhibited symptoms
- No

56. **If yes, what kind of ulcers?**

Check all that apply.

- [ ] Gastric (stomach)
- [ ] Hind gut

57. **If so, how was this horse treated for ulcers?**

Check all that apply.

- [ ] Gastroguard
- [ ] Ulcerguard
- [ ] Sucralfate
- [ ] Ranitidine
- [ ] Succeed hind gut supplement
- [ ] Other hind gut buffer
- [ ] Other:

58. **Has your horse had vitamin E levels measured by your veterinarian?**

Mark only one oval.

- Yes
- No
- Not sure

59. **If yes, what was your horse's vitamin E level**

Mark only one oval.

- Deficient (<1.5 ug/ml)
- Marginal (1.5-2 ug/ml)
- Adequate (>2 ug/ml)
- I'm not sure/can't remember

60. **How would you evaluate your horse's body condition score?**

Mark only one oval.

	1	2	3	4	5	6	7	8	9	
Extremely Thin	◯	◯	◯	◯	◯	◯	◯	◯	◯	Obese

Body Condition Score Chart

Areas of emphasis for body condition scoring: thickening of the neck, fat covering the withers, fat deposits along backbone, fat deposits on flanks, fat deposits on inner thighs, fat deposits around tailhead, fat deposits behind shoulders, fat covering ribs, shoulder blends into neck

Kentucky Equine Research®

859-873-1988, www.ker.com

1 Poor

Animal extremely emaciated; spine, ribs, tailhead, points of hip and buttock projecting prominently; bone structure of withers, shoulders, and neck easily noticeable; no fatty tissue can be felt.

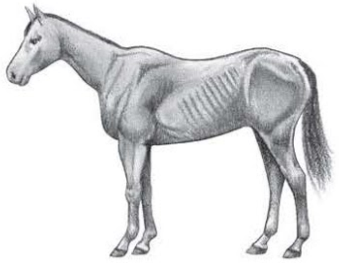

2 Very Thin

Animal emaciated; slight fat covering over base of spine; ribs, tailhead, points of hip and buttock prominent; withers, shoulders, and neck structure faintly discernable.

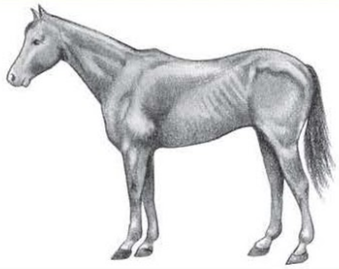

3 Thin

Fat buildup about halfway on spine; slight fat cover over ribs; spine and ribs easily discernable; tailhead prominent, but individual vertebrae cannot be identified visually; points of hip appear rounded but easily discernable; points of buttock not distinguishable; withers, shoulders, and neck accentuated.

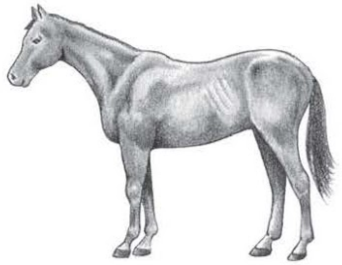

4 Moderately Thin

Slight ridge along back; faint outline of ribs discernable; tailhead prominence depends on conformation, fat can be felt around it; points of hip not discernable; withers, shoulders, and neck not obviously thin.

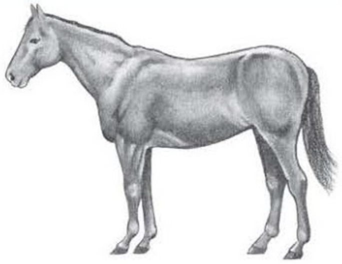

5 Moderate

Back is flat (no crease or ridge); ribs not visually distinguishable but easily felt; fat around tailhead beginning to feel spongy; withers appear rounded over spine; shoulders and neck blend smoothly into body.

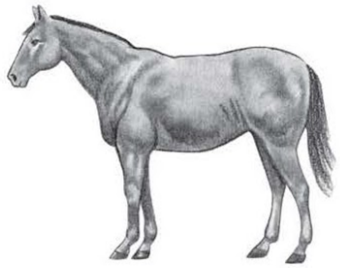

6 Moderately Fleshy

May have slight crease down back; fat over ribs fleshy/spongy; fat around tailhead soft; fat beginning to be deposited along sides of withers, behind shoulders, and along sides of neck.

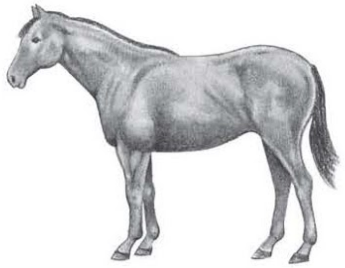

7 Fleshy

May have crease down back; individual ribs can be felt, but noticeable filling between ribs with fat; fat around tailhead soft; fat deposited along withers, behind shoulders, and along neck.

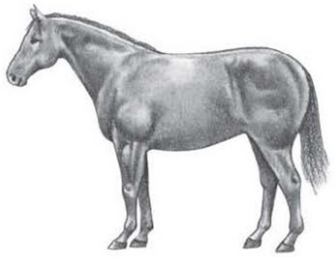

8 Fat

Crease down back; difficult to feel ribs; fat around tailhead very soft; area along withers filled with fat; area behind shoulders filled with fat; noticeable thickening of neck; fat deposited along inner thighs.

9 Extremely Fat

Obvious crease down back; patchy fat appearing.

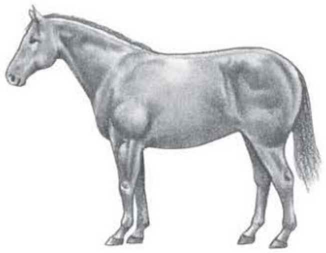

Kentucky Equine Research, 3910 Delaney Ferry Rd., Versailles, KY 40383, 859-873-1988, www.ker.com

© Copyright Kentucky Equine Research, 2010

61. **How would you rate your horse's appetite?**

Consider a rating of 3 as average.
Mark only one oval.

	1	2	3	4	5	
Poor	◯	◯	◯	◯	◯	Superb

62. **Do you consider your horse an easy keeper?**

Mark only one oval.

- Yes, he/she is an easy keeper
- No, my horse maintains weight with the appropriate amount of food
- No, my horse is hard to keep weight on

63. **Does your horse have a "cresty" neck?**

Mark only one oval.

- Yes
- No

64. **Has your horse ever been diagnosed with Equine Metabolic Syndrome or insulin resistance?**

Mark only one oval.

- Yes
- No

65. **Has this horse ever been bred?**

Mark only one oval.

- Yes
- No
- Unsure
